# *Pten* Regulates Cardiomyocyte Differentiation by Modulating Non‐CG Methylation via *Dnmt3*

**DOI:** 10.1002/advs.202100849

**Published:** 2021-07-11

**Authors:** Wuming Wang, Gang Lu, Hong‐Bin Liu, Zhiqiang Xiong, Ho‐Duen Leung, Ruican Cao, Alan Lap‐Yin Pang, Xianwei Su, Patrick Wai Nok Law, Zhiju Zhao, Zi‐Jiang Chen, Wai‐Yee Chan

**Affiliations:** ^1^ CUHK‐SDU Joint Laboratory on Reproductive Genetics School of Biomedical Sciences The Chinese University of Hong Kong Hong Kong China; ^2^ National Research Center for Assisted Reproductive Technology and Reproductive Genetics Jinan 250001 China; ^3^ SDIVF R&D Centre 12W, Hong Kong Science Park Shatin Hong Kong China; ^4^ R&D Division TGD Life Company Limited 15W, Hong Kong Science Park Shatin Hong Kong China

**Keywords:** cardiomyocytes, *Dnmt3*, embryonic stem cells, *Igf2*, non‐CG methylation, *Pten*

## Abstract

The regulation of cardiomyocyte differentiation is a fundamental aspect of cardiac development and regenerative medicine. PTEN plays important roles during embryonic development. However, its role in cardiomyocyte differentiation remains unknown. In this study, a low‐cost protocol for cardiomyocyte differentiation from mouse embryonic stem cells (ESCs) is presented and it is shown that *Pten* deletion potently suppresses cardiomyocyte differentiation. Transcriptome analysis shows that the expression of a series of cardiomyocyte marker genes is downregulated in *Pten^−/−^* cardiomyocytes. *Pten* ablation induces *Dnmt3b* expression via the AKT/FoxO3a pathway and regulates the expression of a series of imprinted genes, including *Igf2*. Double knockout of *Dnmt3l* and *Dnmt3b* rescues the deficiency of cardiomyocyte differentiation of *Pten^−/−^* ESCs. The DNA methylomes from wild‐type and *Pten^−/−^* embryoid bodies and cardiomyocytes are analyzed by whole‐genome bisulfite sequencing. *Pten* deletion significantly promotes the non‐CG (CHG and CHH) methylation levels of genomic DNA during cardiomyocyte differentiation, and the non‐CG methylation levels of cardiomyocyte genes and *Igf2* are increased in *Pten^−/−^* cardiomyocytes. *Igf2* or *Igf1r* deletion also suppresses cardiomyocyte differentiation through the MAPK/ERK signaling pathway, and IGF2 supplementation partially rescues the cardiomyocyte differentiation. Finally, *Pten* conditional knockout mice are generated and the role of PTEN in cardiomyocyte differentiation is verified in vivo.

## Graphical abstract

*Pten* deletion suppresses cardiomyocyte differentiation by reducing the expression of cardiomyocyte genes. A series of imprinted genes are regulated by *Pten*, and *Pten* ablation induces the expression of *Dnmt3* by activating AKT. The non‐CG DNA methylation levels of cardiomyocyte genes are higher in *Pten^−/−^* cardiomyocytes, and *Pten* regulates cardiomyocyte differentiation by modulating non‐CG DNA methylation via *Dnmt3*.

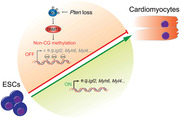

## 1 Introduction

Cardiomyocytes are derived from the mesoderm during embryonic development, and embryonic stem cells (ESCs) can directly differentiate into cardiomyocytes with all of their appropriate structural and functional properties.^[^ ^1^ ^]^ Activin A and BMP4 help to generate highly purified human cardiomyocytes, and the infarcted heart can be repaired with large numbers of cardiomyocytes derived from ESCs.^[^ ^2^ ^]^ Temporal modulation of canonical WNT signaling also contributes to a robust cardiomyocyte differentiation from human pluripotent stem cells.^[^ ^3^ ^]^ In addition, mouse fibroblasts can be transdifferentiated into cardiomyocytes using several transcription factors, such as *Gata4*, *Mef2c*, and *Tbx5*.^[^ ^4^ ^]^ Also, GATA6 and GATA4 are shown to modulate cardiomyocyte hypertrophy,^[^ ^5^ ^]^ and TBX5 and NKX2.5 promote cardiomyocyte differentiation.^[^ ^6^ ^]^ Cardiomyocyte proliferation is regulated by transcription factors, including *Foxp1*.^[^ ^7^ ^]^ Cardiomyocyte‐like cells can also be generated from human fibroblasts upon the treatment of a combination of nine chemical compounds.^[^ ^8^ ^]^

PTEN is a well‐known tumor suppressor that is essential for embryonic development, and PTEN negatively regulates the phosphoinositide‐3 kinase (PI3K) signaling pathway to inhibit AKT activity by dephosphorylating phosphatidylinositol‐3, 4, 5‐trisphosphate.^[^ ^9^ ^]^ The PI3K‐AKT signaling pathway regulates myocardial contractility and cell size, and PTEN can modulate cardiac hypertrophy and survival.^[^ ^10^, ^11^ ^]^ AKT signaling also regulates the proliferation of progenitor cells in the second heart field through the coordination of BMP signaling and *β*‐catenin activity.^[^ ^12^ ^]^ Recently, PTEN is shown to maintain the quiescence of adult muscle stem cells,^[^ ^13^ ^]^ and nuclear PTEN is involved in controlling smooth muscle differentiation by functioning as an indispensable regulator of serum response factor‐dependent transcription.^[^ ^14^ ^]^

Imprinted gene *Igf2* directs cardiomyocyte proliferation during zebrafish heart development and regeneration.^[^ ^15^ ^]^ Imprinted genes are expressed in a parent‐of‐origin‐specific manner. The paternally expressed gene *Igf2* is separated by ≈100 kb from the maternally expressed noncoding gene *H19* on mouse distal chromosome 7, and *Igf2* expression is regulated by the differentially methylated regions (DMRs) in a methylation‐sensitive manner.^[^ ^16^ ^]^ DNA methylation is essential for mammalian development. De novo methylation is mediated by *Dnmt3a* and *Dnmt3b*,^[^ ^17^ ^]^ and DNMT3L cooperates with DNMT3A and DNMT3B to establish imprints.^[^ ^18^, ^19^ ^]^ A loss of *Dnmt3a* and *Dnmt3b* in ESCs obstructs differentiation. For example, *Dnmt3a* loss progressively impairs hematopoietic stem cells differentiation.^[^ ^20^ ^]^ However, the role of these enzymes in somatic stem cells is largely unknown. Cardiomyocyte development, maturation, and disease are orchestrated by dynamic DNA methylation.^[^ ^21^ ^]^ Non‐CG DNA methylation, including CHH and CHG (where H = A, C, or T), has been identified in ESCs and oocytes,^[^ ^22^ ^]^ but is seldom reported in adult somatic cells. Non‐CG methylation is gradually lost upon cell fate specification and is predominantly catalyzed by the *Dnmt3* family.^[^ ^23^ ^]^ Recently, non‐CG DNA methylation was identified in the mouse brain, and both methylated CpGs and CpHs can repress gene transcription in the adult mammalian brain.^[^ ^24^, ^25^ ^]^ DNMT3B was also shown to preferentially mediate non‐CG methylation in the developing heart.^[^ ^26^ ^]^ In our study, we showed that non‐CG DNA methylation occurred in cardiomyocytes and *Pten*‐deficiency induced DNMT3 to promote DNA methylation during cardiomyocyte differentiation.

## 2 Results

### 2.1 Cardiomyocyte Differentiation from ESCs

**Figure 1:**
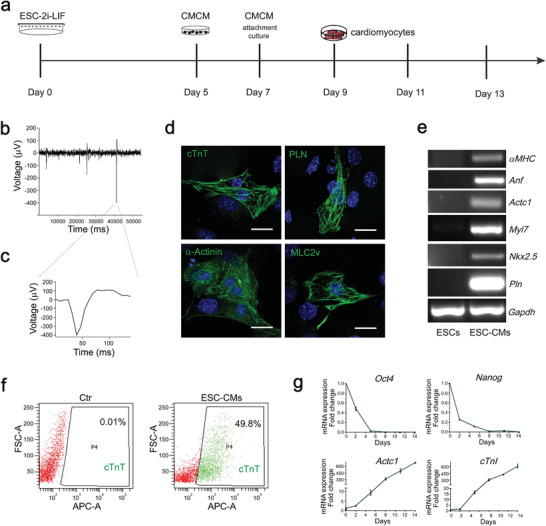
Characterization of ESC‐derived cardiomyocytes. a) Schematic of the cardiomyocyte differentiation protocol from mouse ESCs. b,c) Electrophysiology analysis of the differentiated cardiomyocytes. d) Immunofluorescence of cTnT, PLN, *α*‐Actinin, and MLC2v showed the sarcomeric striations of cardiomyocyte markers. Scale bars, 20 µm. e) RT‐PCR analysis of cardiac marker gene expression in ESCs and ESC‐CMs. f) Flow cytometry provided a quantitative method to evaluate the relative yield and purity of cardiomyocytes by measuring the number of cTnT‐positive cells. g) Q‐PCR analysis of pluripotency markers (*Oct4* and *Nanog*) and cardiomyocyte markers (*Actc1* and *cTnI*) from day 0 to day 14 of cardiomyocyte differentiation.

Directed differentiation of cardiomyocytes from induced pluripotent stem cells and ESCs constitutes a cell source for heart development modeling and drug screening. Canonical WNT signaling is required for the development of cardiac progenitors and is a positive regulator of cardiac progenitor proliferation.^[^ ^27^ ^]^ The combination of Activin A and BMP4 promotes cardiac differentiation of mouse and human pluripotent stem cells.^[^ ^28^ ^]^ Here, we present a protocol to direct ESCs into functional cardiomyocytes in a growth factor‐free system. The mouse ESCs were cultivated in vitro as 3D aggregates called embryoid bodies (EBs) for 5 days in ESC culture medium without 2i (2i, PD0325901 and CHIR99021) and LIF (leukemia inhibitory factor), and the EBs were transferred to suspension culture for further differentiation with cardiomyocyte culture medium (CMCM). On day 7, the EBs were transferred to attachment culture in tissue culture dishes with CMCM (**Figure** **1**a). ESC‐derived cardiomyocytes (ESC‐CMs) started to beat spontaneously on day 9 (Figure 1a; Video S1, Supporting Information) and displayed spontaneous electrophysiological activity in a microelectrode array experiment (Figure 1b,c). The ESC‐CMs expressed cardiac troponin T (cTnT), phospholamban (PLN), *α*‐Actinin, and MLC2v and exhibited sarcomeric striations (Figure 1d; Figure S1, Supporting Information). We also examined the mRNA level of a series of cardiac‐specific markers by RT‐PCR, and there was a significant increase in the expression of these markers in day‐10 ESC‐CMs when comparing with ESCs (Figure 1e). The ESC‐CMs were analyzed by flow cytometry, and 49.8% of these cells were cTnT‐positive (Figure 1f). We measured the expression of pluripotency genes (*Oct4* and *Nanog*) and cardiomyocyte markers (*Actc1* and *cTnI*) at different time points during cardiomyocyte differentiation. As expected, the pluripotency gene expression was decreased, while the expression of cardiomyocyte markers was increased (Figure 1g). These results indicate that the cardiac differentiation from mouse ESCs was efficient under this protocol.

### 2.2 *Pten* Deficiency Suppresses Cardiomyocyte Differentiation

**Figure 2:**
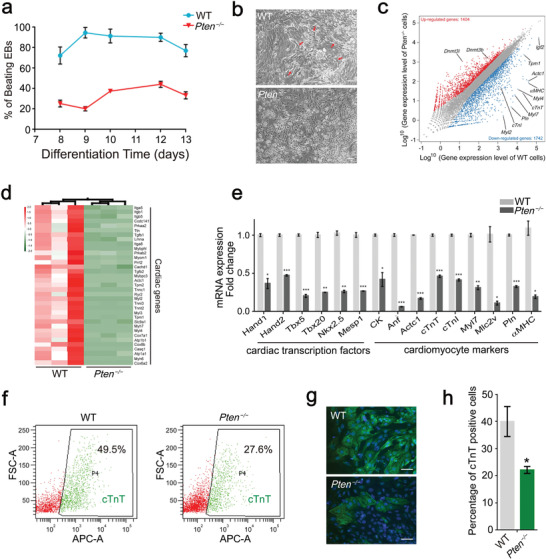
*Pten* ablation suppresses the cardiomyocyte differentiation. a) Beating rates of EBs derived from WT and *Pten^−/−^* ESCs at different time points. Error bars indicate the mean ± SD (*n* = 3). b) Phase‐contrast images of WT and *Pten^−/−^* cardiomyocytes. Beating cardiomyocytes are indicated with red arrows in the WT group. Scale bars, 100 µm. c) Scatter plot of transcript expression in WT and *Pten^−/−^* cardiomyocytes. Expression values are shown on a log^10^ scale. Red dots indicate upregulated genes in *Pten^−/−^* cardiomyocytes, and blue dots indicate downregulated genes. d) Heat map of FPKM values of a series of cardiac genes in WT and *Pten^−/−^* cardiomyocytes. The heat map was normalized with sigma‐normalization per row. See also Table S2, Supporting Information. e) The expression of cardiac transcription factors and cardiomyocyte markers was assessed by Q‐PCR in WT and *Pten^−/−^* cardiomyocytes. Error bars indicate the mean ± SD (*n* = 3). *p* values were calculated by the Student's *t*‐test: *p < 0.05; **p < 0.01; and ***p < 0.001. f) Representative flow cytometry analysis of the proportion of cTnT‐positive cells among WT and *Pten^−/−^* cardiomyocytes. g) Representative immunofluorescence images of cardiomyocytes stained for cTnT. Scale bars, 50 µm. h) The proportion of cTnT‐positive cells in WT and *Pten^−/−^* cardiomyocytes. Error bars indicate the mean ± SD (*n* = 3). *p* value was calculated by the Student's *t*‐test: *p < 0.05.

PTEN is a tumor suppressor that negatively regulates the PI3K/AKT signaling pathway, and PTEN can regulate cardiac hypertrophy and survival.^[^ ^10^ ^]^ To explore the function of *Pten* in cardiomyocyte differentiation, we generated *Pten*‐deficient mouse ESCs using the CRISPR‐Cas9 system as described previously.^[^ ^29^ ^]^ Cardiomyocytes were generated from wild‐type (WT) and *Pten^−/−^* ESCs using the differentiation protocol described above. Intriguingly, *Pten^−/−^* EBs had a lower spontaneous beating rate than WT EBs (**Figure** **2**a; Videos S2,S3, Supporting Information). The cells that differentiated from *Pten^−/−^* ESCs tended to be neural stem cell‐like cells, which is consistent with our previous findings that *Pten* loss promoted neural ectoderm differentiation,^[^ ^29^ ^]^ and WT ESCs could differentiate into spontaneously beating cardiomyocytes (Figure 2b; Videos S4,S5, Supporting Information).

We used RNA sequencing (RNA‐seq) to analyze the transcriptomes of cardiomyocytes derived from WT and *Pten^−/−^* ESCs, and the enrichment of cardiac disease and development related signaling pathways was significant based on the analyses from the Kyoto Encyclopedia of Genes and Genomes (KEGG)‐differential expression gene (DEG) relationship network, the Enriched KEGG Pathway, and the functional pathway enrichment of DEGs (Figure S2a–c, Supporting Information). A pairwise comparison indicated that 1404 genes were significantly upregulated in *Pten^−/−^* cardiomyocytes, and 1742 genes were significantly downregulated, including the mature cardiomyocyte markers such as *cTnT*, *Myl2*, *cTnI*, *Pln*, *Myl7*, *Tpm1*, *Myl4*, *αMHC*, and *Actc1* (Figure 2c). As indicated in Figure 2d, there was a significant difference in the expression level of a series of cardiac genes (including cardiac muscle contraction markers) between the WT and *Pten^−/−^* group, which may explain the lower beating rate in *Pten^−/−^* EBs (Figure 2a; Videos S2,S3, Supporting Information). To verify the expression of cardiac‐specific markers, we measured the mRNA levels of cardiac transcription factors and cardiomyocyte markers in WT and *Pten^−/−^* EBs by Q‐PCR; the expression of all tested marker genes was found to be decreased in *Pten^−/−^* EBs (Figure 2e). cTnT is well known to be indispensable in sarcomere assembly and cardiac contractility.^[^ ^30^ ^]^ The differentiated cardiomyocytes were analyzed by flow cytometry for the presence of cTnT. As expected, the proportion of cTnT‐positive cells decreased significantly in the *Pten^−/−^* group when compared with the WT group (Figure 2f). This observation was further confirmed by an immunofluorescence staining of cTnT‐positive cells (Figure 2g,h). Our data indicate that *Pten* ablation suppresses the differentiation of cardiomyocyte from ESCs.

To verify the role of *Pten* in cardiomyocyte differentiation, *Pten* was overexpressed in *Pten^−/−^* ESCs using a lentivirus system (Figure S3a, Supporting Information), and the *Pten*‐overexpressing (*Pten* OE) ESCs were differentiated into cardiomyocytes. *Pten* OE restored the expression of cardiomyocyte marker genes (Figure S3b, Supporting Information), as well as the proportion of cTnT‐positive cells (Figure S3c, Supporting Information). Phosphorylation of the C‐terminal of PTEN at S380, T382, and T383 is shown to inhibit its phosphatase activity.^[^ ^31^ ^]^ We utilized the PTEN‐A3 mutant (S380A, T382A, and T383A) ESC line described previously to augment the phosphatase activity,^[^ ^29^ ^]^ and the cardiomyocytes derived from PTEN‐A3 mutant ESCs displayed a higher expression level of cardiomyocyte genes (Figure S3d, Supporting Information). In addition, we measured the level of PTEN expression at different time points during cardiomyocyte differentiation and found that PTEN expression was potently induced on day 5 and decreased in the following days (Figure S3e, Supporting Information). This indicates that *Pten* may be involved in the induction of cardiomyocyte differentiation at an early stage. In conclusion, the loss of *Pten* strongly suppresses the differentiation of cardiomyocytes from ESCs.

### 2.3 *Pten* Ablation Modulates the Expression of Imprinted Genes and Induces DNMT3L and DNMT3B

**Figure 3:**
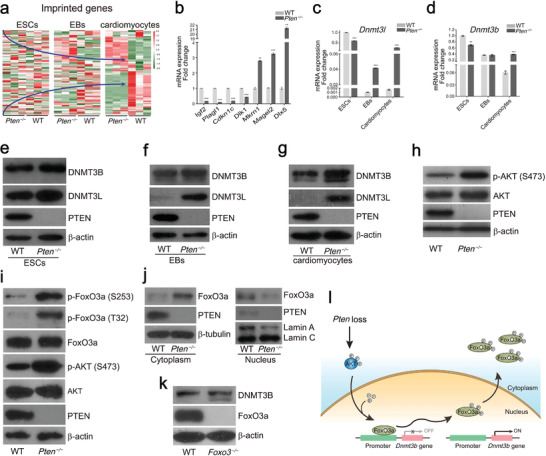
*Pten* deletion affects the expression of DNMT3 via AKT/FoxO3a signaling pathway. a) Heat map of FPKM values of imprinted genes in WT and *Pten^−/−^* ESCs, EBs, and cardiomyocytes. See also Table S3, Supporting Information. b) Q‐PCR analysis of mRNA expression of imprinted genes (*Igf2*, *Plagl1*, *Cdkn1c*, *Dlk1*, *Mkrn1*, *Magel2*, and *Dlx5*). Error bars indicate the mean ± SD (*n* = 3). *p* values were calculated by the Student's *t*‐test: **p < 0.01 and ***p < 0.001. c) Q‐PCR analysis of mRNA expression of *Dnmt3l* in WT and *Pten^−/−^* ESCs, EBs, and cardiomyocytes. Error bars indicate the mean ± SD (*n* = 3). *p* values were calculated by the Student's *t*‐test: ***p < 0.001. d) Q‐PCR analysis of mRNA expression of *Dnmt3b* in WT and *Pten^−/−^* ESCs, EBs, and cardiomyocytes. Error bars indicate the mean ± SD (*n* = 3). *p* values were calculated by the Student's *t*‐test: **p < 0.01 and ***p < 0.001. e) Protein levels of DNMT3B and DNMT3L in WT and *Pten^−/−^* ESCs. f) Protein levels of DNMT3B and DNMT3L in WT and *Pten^−/−^* EBs. g) Protein levels of DNMT3B and DNMT3L in WT and *Pten^−/−^* cardiomyocytes. h) Phosphorylation of AKT at S473 in WT and *Pten^−/−^* cardiomyocytes. i) Phosphorylation levels of FoxO3a at S253 and T32 in WT and *Pten^−/−^* cardiomyocytes. j) Protein levels of FoxO3a in the cytoplasm and nucleus of WT and *Pten^−/−^* cardiomyocytes. k) Protein level of DNMT3B in WT and *Foxo3^−/−^* cardiomyocytes. l) A model for how *Pten* loss promotes the expression of *Dnmt3b* via AKT/FoxO3a signaling pathway.

To understand why the loss of *Pten* suppresses cardiomyocyte differentiation, we compared the transcriptomes of WT and *Pten^−/−^* ESCs, EBs, and cardiomyocytes. Upon the induction of cardiomyocyte differentiation from ESCs, the expression of a series of imprinted genes was progressively affected by the absence of *Pten* (**Figure** **3**a). The change in expression pattern of these genes (as indicated by the arrows) becomes prominent toward the completion of differentiation. To verify this observation, we analyzed the imprinted gene transcript levels by Q‐PCR and found that the levels of *Igf2*, *Plagl1*, *Cdkn1c*, and *Dlk1* were significantly decreased in the *Pten^−/−^* group, whereas the transcript levels of *Mkrn1*, *Magel2*, and *Dlx5* were significantly increased (Figure 3b).

DNMT3L cooperates with DNMT3A and DNMT3B to establish maternal genomic imprints in mice.^[^ ^18^, ^32^ ^]^ Most notably, we found that the level of *Dnmt3l* and *Dnmt3b* transcripts was significantly elevated in *Pten^−/−^* cardiomyocytes (Figures 2c and 3c,d). To further verify this finding, we compared the protein levels of DNMT3L and DNMT3B in WT and *Pten^−/−^* ESCs, EBs, and cardiomyocytes. The protein levels of both DNMT3L and DNMT3B were higher in *Pten^−/−^* cardiomyocytes, while the there was no difference between WT and *Pten^−/−^* ESCs (Figure 3e,g). Upon differentiation from WT ESCs, the expression of DNMT3L was downregulated in EBs and cardiomyocytes (Figure 3c and 3e–g), while the level of DNMT3L was maintained in *Pten^−/−^* groups (Figure 3f,g). In addition, there was no significant difference in the expression of DNMT3B between WT and *Pten^−/−^* EBs (Figure 3d,f). It suggests that the expression of *Dnmt3l* and *Dnmt3b* was not induced by *Pten* deletion at the same time point. The inactivation of AKT results in the downregulation of DNMT1 and DNMT3B levels,^[^ ^33^ ^]^ and *Pten* ablation potently promoted the AKT activity (Figure 3h). AKT lies at a signaling node downstream of PI3K. To further verify the relationship of AKT and DNMT3, we evaluated the effect of PI3K inhibitor (PX‐866) on DNMT3 expression in *Pten^−/−^* cells. Our result showed that PI3K inhibitor significantly abated the expression of DNMT3 by inhibiting AKT activity (Figure S4a, Supporting Information). We also evaluated the protein level of DNMT3 in PTEN‐A3 mutant cells and found that the PTEN‐A3 mutation significantly suppressed the expression of DNMT3L and DNMT3B by inhibiting AKT activity (Figure S4b, Supporting Information). Recently, FoxO3A was shown to negatively regulate *Dnmt3b* expression by interacting with the binding element *Foxo3a* (+166–+173) of *Dnmt3b* promoter. An overexpression of FoxO3a or a combined treatment with doxorubicin induces an accumulation of FoxO3a in the nucleus to suppress *Dnmt3b* expression.^[^ ^34^, ^35^ ^]^ AKT‐dependent phosphorylation of FoxO3a at Thr32 and Ser253 promotes the translocation of FoxO3a from the nucleus to the cytoplasm.^[^ ^36^ ^]^ In our study, the phosphorylation of FoxO3a at Thr32 and Ser253 was induced by AKT in *Pten^−/−^* group (Figure 3i), and the phosphorylation triggered the translocation of FoxO3a from the nucleus to cytoplasm (Figure 3j). To verify that *Dnmt3b* was directly regulated by FoxO3a, we deleted *Foxo3* gene with CRISPR‐Cas9 system and measured the expression of DNMT3B in WT and *Foxo3^−/−^* cardiomyocytes. As expected, the DNMT3B level was elevated in *Foxo3^−/−^* group (Figure 3k). We also utilized doxorubicin to induce FoxO3a nuclear accumulation, and the DNMT3B was significantly suppressed by doxorubicin (Figure S4c,d, Supporting Information). We concluded that *Pten* deletion promoted the activity of AKT to phosphorylate FoxO3a at Thr32 and Ser253, which triggered the translocation of FoxO3a from the nucleus to cytoplasm and derepressed the inhibition of *Dnmt3b* promoter (Figure 3l).

We generated *Pten^−/−^/Dnmt3l^−/−^* ESCs using the CRISPR‐Cas9 system (Figure S5a, Supporting Information). The mRNA expression of the cardiac marker genes was significantly increased in *Pten^−/−^/Dnmt3l^−/−^* cells when compared with *Pten^−/−^* cells (Figure S5b, Supporting Information), and the proportion of cTnT‐positive cells was elevated in the absence of *Dnmt3l^−/−^* (Figure S5c, Supporting Information). This result indicates that *Dnmt3l* deletion partially rescues the defective cardiomyocyte differentiation of *Pten^−/−^* ESCs. Furthermore, we generated *Pten^−/−^/Dnmt3l^−/−^/Dnmt3b^−/−^* ESCs with the CRISPR‐Cas9 system (Figure S5d, Supporting Information). There were more cardiomyocytes in the *Dnmt3l^−/−^/Dnmt3b^−/−^* double knockout group when compared with the *Pten^−/−^* group (Figure S5e, Supporting Information). Flow cytometry also showed that *Dnmt3l^−/−^/Dnmt3b^−/−^* double deletion could fully rescue the proportion of cTnT‐positive cells (Figure S5f, Supporting Information). The mRNA levels of a series of cardiomyocyte markers in the *Pten^−/−^/Dnmt3l^−/−^/Dnmt3b^−/−^* group resembled those found in the WT group (Figure S5g, Supporting Information). On the other hand, the overexpression of *Dnmt3b/Dnmt3l* inhibits the cardiomyocyte differentiation by reducing the proportion of cTnT‐positive cells and suppressing the expression of cardiac genes; these results are similar to the phenotype induced by *Pten^−/−^* (Figure S5h–j, Supporting Information). We thus concluded that DNMT3 might be the downstream effector of the PTEN/AKT signaling pathway in regulating cardiomyocyte differentiation.

### 2.4 *Pten* Ablation Promotes Non‐CG Methylation during Cardiomyocyte Differentiation

**Figure 4:**
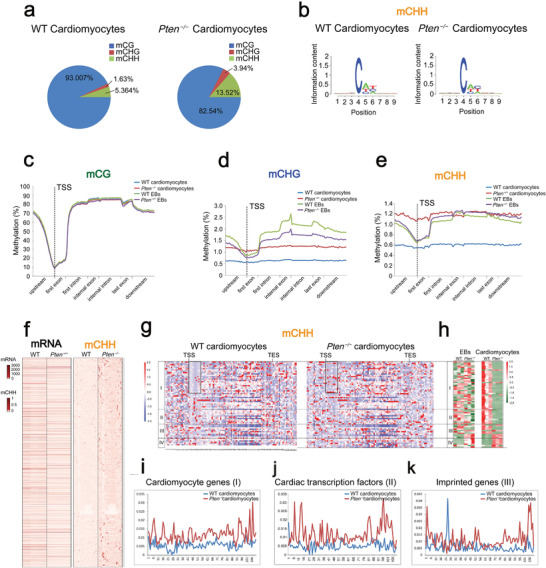
The genomic DNA methylation landscape in WT and *Pten^−/−^* cardiomyocytes. a) Pie chart showing the percentages of total methylcytosine events that occur in the contexts of CG, CHG, and CHH in WT and *Pten^−/−^* cardiomyocytes. b) Sequence logos are shown for bases proximal to hypermethylated CHHs in WT and *Pten^−/−^* cardiomyocytes. c–e) The average methylation levels for CG, CHG, and CHH are shown along the upstream, first exon, first intron, internal exon, internal intron, last exon, and downstream regions for all genes in WT and *Pten^−/−^* cardiomyocytes. See also Table S4, Supporting Information. f) Heat map showing the mRNA and mCHH levels of DEGs of WT and *Pten^−/−^* cardiomyocytes. See also Tables S5 and S6, Supporting Information. g,h) Heat map of CHH methylation and mRNA levels of cardiomyocyte genes, cardiac transcription factors, imprinted genes, and pluripotency genes in *Pten^−/−^* cardiomyocytes compared to WT cardiomyocytes. I, cardiomyocyte genes; II, cardiac transcription factors; III, imprinted genes; IV, pluripotency genes. See also Tables S7 and S8, Supporting Information. i–k) The genomic distribution of CHH methylation of cardiomyocyte genes, cardiac transcription factors, and imprinted genes in WT and *Pten^−/−^* cardiomyocytes.

The ESC pluripotency is associated with a global DNA hypomethylation,^[^ ^37^ ^]^ and the methylome undergoes dynamic changes during differentiation.^[^ ^38^ ^]^ In addition, the dynamic changes in DNA methylation control gene expression in cardiomyocyte development, maturation, and disease.^[^ ^21^, ^39^ ^]^ The surprising observation that *Pten* ablation induced DNMT3B and DNMT3L expression and affected the expression of imprinted genes raised a question of whether the suppressed cardiomyocyte differentiation was caused by an induction of DNA methyltransferase. To investigate the change in DNA methylomes during cardiomyocyte differentiation, we performed whole‐genome bisulfite sequencing (WGBS) of WT and *Pten^−/−^* EBs as well as cardiomyocytes. Intriguingly, *Pten* deletion significantly promoted the proportion of non‐CG (CHG and CHH) methylation in cardiomyocytes (**Figure** **4**a), and the methylation level distribution showed that the percentage of mCHG, mCHH, and mC at the 20% methylation level was elevated in *Pten^−/−^* cardiomyocytes (Figure S6a, Supporting Information).

The DNA sequences around methylated CHGs in EBs showed a strong enrichment of motifs that largely resembled those found in ESCs.^[^ ^24^, ^40^ ^]^ During cardiomyocyte differentiation via EBs (Figure S6b, Supporting Information), the sequence preferences for CHG contexts in cardiomyocytes were similar to those in EBs. However, the CHH contexts varied between EBs and cardiomyocytes, with EBs preferred CAC and cardiomyocytes preferred CAT (Figure S6c,d, Supporting Information). There was also a slight difference in the sequence preferences for CHH contexts between WT and *Pten^−/−^* cardiomyocytes, with WT cardiomyocytes preferred CAT and *Pten^−/−^* cardiomyocytes preferred CAC (Figure 4b).

Next, we examined the genomic distribution of mCG, mCHG, and mCHH in WT and *Pten^−/−^* EBs and cardiomyocytes. mCG was less present at transcription start sites (TSSs), and there was no significant difference in CG methylation level in the genic, upstream, or downstream regions of WT and *Pten^−/−^* EBs and cardiomyocytes (Figure 4c). Intriguingly, the *Pten^−/−^* EBs had significantly reduced methylation in the context of CHG compared to WT EBs, while *Pten^−/−^* cardiomyocytes showed higher methylation than WT cardiomyocytes in the context of CHG (Figure 4d). In addition, the methylation level in the context of CHG in EBs was higher than that in cardiomyocytes (Figure 4d), indicating that the level of CHG methylation decreases as cardiomyocytes differentiate. The CHH methylation was slightly higher in *Pten^−/−^* EBs compared with WT EBs from the first intron to the downstream region, and the *Pten^−/−^* cardiomyocytes showed a significantly higher CHH methylation than WT cardiomyocytes (Figure 4e).

We analyzed the DNA methylation level in the context of mCHH within the genic regions in more detail and the mRNA level of all DEGs. In general, the mCHH level in *Pten^−/−^* cells was higher than that of the WT cells (Figure 4f). Specially, we analyzed the mCHH and mRNA levels of cardiomyocyte genes (I), cardiac transcription factors (II), imprinted genes (III), and pluripotency genes (IV). The first exons of 24 cardiomyocyte marker genes were demethylated in WT cardiomyocytes, while the *Pten^−/−^* group was methylated in these regions (the bracketed regions in Figure 4g). The mRNA level of the cardiomyocyte genes was downregulated in *Pten^−/−^* cardiomyocytes when compared with the WT counterparts, and the difference was not significant between WT and *Pten^−/−^* EBs (Figure 4h). Thus, we concluded that the genic non‐CG methylation was negatively correlated with cardiomyocyte gene expression (Figure 4g,h; Figure S6e, Supporting Information). We also compared the average CHH methylation levels of genic regions of cardiomyocyte genes, cardiac transcription factors, and imprinted genes: *Pten* ablation significantly increased the mCHH levels (Figure 4i–k). In addition, we analyzed the dynamic DNA methylation in the context of mCHG within the genic regions in more detail (Figure S6f,g, Supporting Information) and compared the average CHG methylation levels of the genic regions of cardiomyocyte genes, cardiac transcription factors, and imprinted genes between WT and *Pten^−/−^* cardiomyocytes (Figure S6h–j, Supporting Information). An observation similar to the case of mCHH was obtained. Taken together, our results suggested that the regulation of non‐CG methylation by *Pten* plays an important role in cardiac gene and imprinted gene programming during cardiomyocyte differentiation.

**Figure 5:**
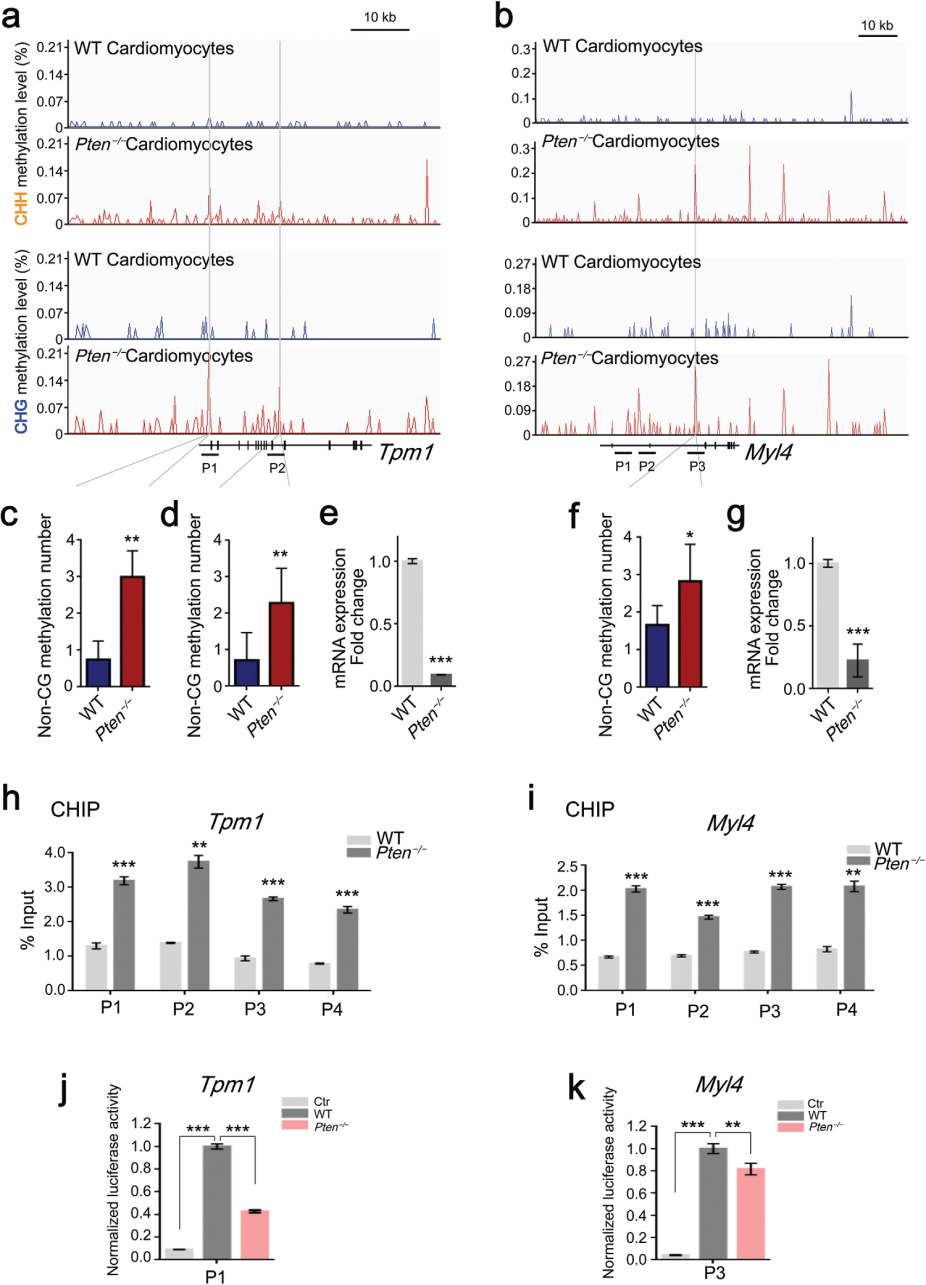
Repression of cardiac genes by non‐CG methylation. a,b) IGV traces of CHG and CHH methylation of *Tpm1* and *Myl4* in WT and *Pten^−/−^* cardiomyocytes. c,d) Gene body methylation of the *Tpm1* gene in WT and *Pten^−/−^* cardiomyocytes by bisulfite sequencing PCR. Error bars indicate the mean ± SD (*n* = 7). *p* values were calculated by the Student's *t*‐test: **p < 0.01. e) Gene expression of *Tpm1* in WT and *Pten^−/−^* cardiomyocytes. Error bars indicate the mean ± SD (*n* = 3). *p* value were calculated by the Student's *t*‐test: ***p < 0.001. f) Gene body methylation of the *Myl4* gene in WT and *Pten^−/−^* cardiomyocytes by bisulfite sequencing PCR. Error bars indicate the mean ± SD (*n* = 6). *p* value were calculated by the Student's *t*‐test: *p < 0.05. g) Gene expression of *Myl4* in WT and *Pten^−/−^* cardiomyocytes. Error bars indicate the mean ± SD (*n* = 3). *p* value was calculated by the Student's *t*‐test:***p < 0.001. h,i) qPCR analysis of ChIP experiments on the indicated genomic regions in WT and *Pten^−/−^* cardiomyocytes. Error bars indicate the mean ± SD (*n* = 3). *p* values were calculated by the Student's *t*‐test: **p < 0.01 and ***p < 0.001. j,k) Luciferase reporter assay of *Tpm1* and *Myl4* promoters of WT and *Pten^−/−^* cardiomyocytes. Error bars indicate the mean ± SD (*n* = 3). *p* values were calculated by the Student's *t*‐test: **p < 0.01 and ***p < 0.001.

To further verify this notion, a visual inspection of the Integrated Genomics Viewer (IGV) traces of cardiomyocyte genes was performed. In the case of *Tpm1* and *Myl4*, de novo non‐CG methylation of both genes was observed in *Pten^−/−^* cardiomyocytes (**Figure** **5**a,b). We analyzed the non‐CG methylation level of the gene bodies by bisulfite sequencing PCR and found that *Pten* deficiency significantly promoted the non‐CG methylation of *Tpm1* and *Myl4* (Figure 5c,d,f). Non‐CG methylation was inversely correlated with the expression of *Tpm1* and *Myl4* (Figure 5e,g). Chromatin immunoprecipitation (ChIP)‐qPCR studies showed that DNMT3B binds to the *Tpm1* and *Myl4* gene bodies containing non‐CG methylation sequence, and *Pten* deletion promoted the binding of DNMT3B to non‐CG methylation sequence (Figure 5h,i). In addition, we analyzed the luciferase activity of promoter sequences of the two genes, with non‐CG methylation sequences included, from WT and *Pten^−/−^* cardiomyocytes; our results showed that the luciferase activity was decreased in the *Pten^−/−^* groups (Figure 5j,k). Taken together, these data demonstrated that *Pten*‐regulates non‐CG methylation to affect cardiomyocyte gene expression.

### 2.5 The Imprinted Gene *Igf2* Is Regulated by Dynamic Methylation and Is Involved in Cardiomyocyte Differentiation

**Figure 6:**
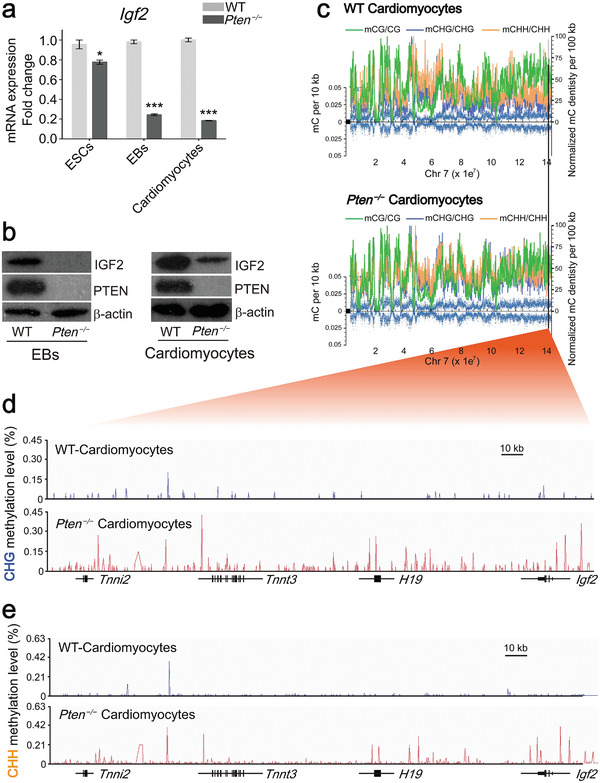
The imprinted gene *Igf2* was affected by *Pten* through non‐CG methylation. a) Q‐PCR analysis of mRNA expression of *Igf2* in WT and *Pten^−/−^* ESCs, EBs, and cardiomyocytes. Error bars indicate the mean ± SD (*n* = 3). *p* values were calculated by the Student's *t*‐test: *p < 0.05 and ***p < 0.001. b) Protein levels of IGF2 in WT and *Pten^−/−^* EBs and cardiomyocytes. c) A chromosome‐wide (chr7) view of DNA methylation (mCG, mCHG, and mCHH) for WT and *Pten^−/−^* cardiomyocytes. d,e) IGV traces of CHG and CHH methylation of *Igf2* in WT and *Pten^−/−^* cardiomyocytes.

The imprinted expression of *Igf2* is controlled by the methylation of a CTCF‐dependent boundary,^[^ ^41^ ^]^ and cardiomyocyte differentiation is regulated by growth factors such as IGF2 and FGF2. FGF2 controls the differentiation of resident cardiac precursors into functional cardiomyocytes.^[^ ^42^ ^]^ IGF signaling directs ventricular cardiomyocyte proliferation during embryonic heart development,^[^ ^43^ ^]^ and IGF promotes cardiac lineage induction in vitro by selective expansion of early mesoderm.^[^ ^44^ ^]^ We found that the expression of *Igf2* was reduced progressively by *Pten* ablation as ESCs differentiated into cardiomyocytes (Figures 2c and 6a), and the protein level of IGF2 was also decreased by *Pten* deletion in EBs and cardiomyocytes (**Figure** **6**b). We examined the IGF2 protein level in culture medium by the enzyme‐linked immunosorbent assay (ELISA), and there were higher levels of IGF2 protein in the WT group than the *Pten^−/−^* group at different time points during cardiomyocyte differentiation (Figure S7a, Supporting Information). Thus, we hypothesized that *Igf2* is involved in the regulation of cardiomyocyte differentiation by *Pten*.

A global‐scale view of the methylated cytosine density showed that the density of non‐CG DNA methylation varied throughout Chromosome 7 between WT and *Pten^−/−^* cardiomyocytes (Figure 6c). Visual inspection of the IGV traces of the imprinted genes *Igf2* and *H19* identified an increase in non‐CG methylation (CHG and CHH) in *Pten^−/−^* cardiomyocytes when compared with WT cardiomyocytes (Figure 6d,e). These results suggested that the expression of the imprinted gene *Igf2* was correlated with the dynamic non‐CG methylation level during cardiomyocyte differentiation.

**Figure 7:**
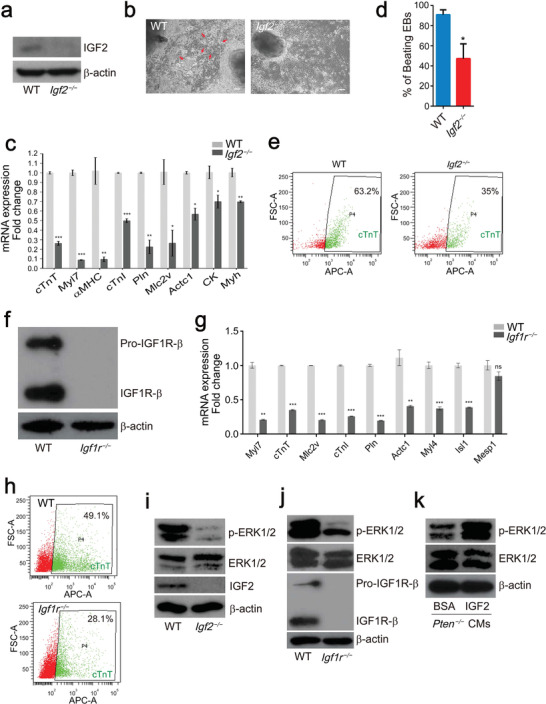
*Igf2* or *Igf1r* ablation suppressed cardiomyocyte differentiation. a) Western blot analysis of the protein level of IGF2 in WT and *Igf2^−/−^* cardiomyocytes. b) Phase‐contrast images of WT and *Igf2^−/−^* cardiomyocytes. Differentiated cardiomyocytes are indicated with red arrows in the WT group. Scale bars, 100 µm. c) Q‐PCR analysis of the mRNA expression of cardiomyocyte marker genes in WT and *Igf2^−/−^* cardiomyocytes. Error bars indicate the mean ± SD (*n* = 3). *p* values were calculated by the Student's *t*‐test: *p < 0.05, **p < 0.01, and ***p < 0.001. d) Beating rate of EBs derived from WT and *Igf2^−/−^* ESCs. Error bars indicate the mean ± SD (*n* = 3). *p* value was calculated by the Student's *t*‐test: *p < 0.05. e) Representative flow cytometry analysis of the proportion of cTnT‐positive cells in WT and *Igf2^−/−^* cardiomyocytes. f) Western blot analysis of the protein level of IGF1R in WT and *Igf1r^−/−^* cardiomyocytes. g) Q‐PCR analysis of the mRNA expression of cardiomyocyte marker genes in WT and *Igf1r^−/−^* cardiomyocytes. Error bars indicate the mean ± SD (*n* = 3). *p* values were calculated by the Student's *t*‐test: **p < 0.01 and ***p < 0.001. h) Representative flow cytometry analysis of the proportion of cTnT‐positive cells in WT and *Igf1r^−/−^* cardiomyocytes. i) ERK1/2 activity in WT and *Igf2^−/−^* cardiomyocytes. j) ERK1/2 activity in WT and *Igf1r^−/−^* cardiomyocytes. k) ERK1/2 activity in *Pten^−/−^* cardiomyocytes treated with bovine serum albumin (BSA) and IGF2.

To verify the role of *Igf2* in cardiomyocyte differentiation regulated by PTEN, we deleted the *Igf2* gene in WT ESCs using the CRISPR‐Cas9 system, and IGF2 protein could not be detected by Western blot (**Figure** **7**a). *Igf2* deletion significantly suppressed cardiomyocyte differentiation (Figure 7b). WT ESCs were differentiated to beating cardiomyocytes as indicated with red arrows, while *Igf2^−/−^* ESCs were differentiated into neural stem cell‐like cells, which was similar to the *Pten^−/−^* group (Figures 2b and 7b). The expression of cardiomyocyte markers was decreased in *Igf2^−/−^* cardiomyocytes (Figure 7c), and the beating rate of EBs was also suppressed in the *Igf2^−/−^* group (Figure 7d). We dissociated the cultured cells and performed flow cytometry for cTnT, and the *Igf2^−/−^* group displayed a lower percentage of cTnT‐positive cells than WT cells (Figure 7e). In addition, we cultured *Pten^−/−^* cells in the presence of IGF2 in the culture medium during cardiomyocyte differentiation. As expected, IGF2 supplementation promoted the expression of cardiomyocyte markers such as *cTnT*, *Myl7*, *αMHC*, *cTnI*, *Mlc2a*, and *Pln* (Figure S7b, Supporting Information).

IGF1R is a receptor of IGF2, and IGF2/IGF1R signaling is known to activate the PI3K/AKT and MAPK/ERK signaling pathway.^[^ ^45^ ^]^ We generated *Igf1r^−/−^* ESCs (Figure 7f), and the cardiomyocyte differentiation was also suppressed by *Igf1r* deletion (Figure 7g). Flow cytometry also showed a lower proportion of cTnT‐positive cells in the *Igf1r^−/−^* group (Figure 7h). These results indicated that *Igf2* promoted cardiomyocyte differentiation through *Igf1r*. To verify the regulatory mechanism, we measured AKT activities in the WT and *Igf2^−/−^* cells at different time points during cardiomyocyte differentiation. While no noticeable difference was observed at the initial stage (Day 0 and Day 3), the AKT activity was significantly decreased in *Igf2^−/−^* cells on Day 5 and Day 7 (Figure S7c, Supporting Information). Despite PTEN expression was induced at the same initial stage in WT cells (Figure S3e, Supporting Information), and the AKT activity was continually activated by *Pten* deletion (Figure S7d, Supporting Information). These results suggested that IGF2/IGF1R may enhance cardiomyocyte differentiation through signaling pathways other than PI3K/AKT. Extracellular signal‐regulated kinase 1/2 (ERK1/2) signaling is a key pathway in regulating cardiac development and likely occupies a central regulatory position in the signaling hierarchy of cardiac myocytes.^[^ ^46^ ^]^ IGF2 signaling directs ventricular cardiomyocyte proliferation by activating ERK signaling.^[^ ^47^ ^]^ Thus, we measured the MAPK/ERK signaling in the WT and *Igf2^−/−^* group, and the phosphorylation of ERK1/2 was significantly decreased in the *Igf2^−/−^* group (Figure 7i). This was consistent with the results in the WT and *Igf1r^−/−^* groups (Figure 7j). In contrast, the presence of IGF2 in the culture medium could also induce ERK activity (Figure 7k). In the results presented above, we concluded that DNMT3 expression might be regulated by the PTEN/AKT pathway (Figure 3h; Figure S4a, Supporting Information). Here we conclude that IGF2 might augment cardiomyocyte differentiation through the MAPK/ERK signaling pathway.

### 2.6 PTEN's Roles in Regulating Cardiac Development In Vivo

**Figure 8:**
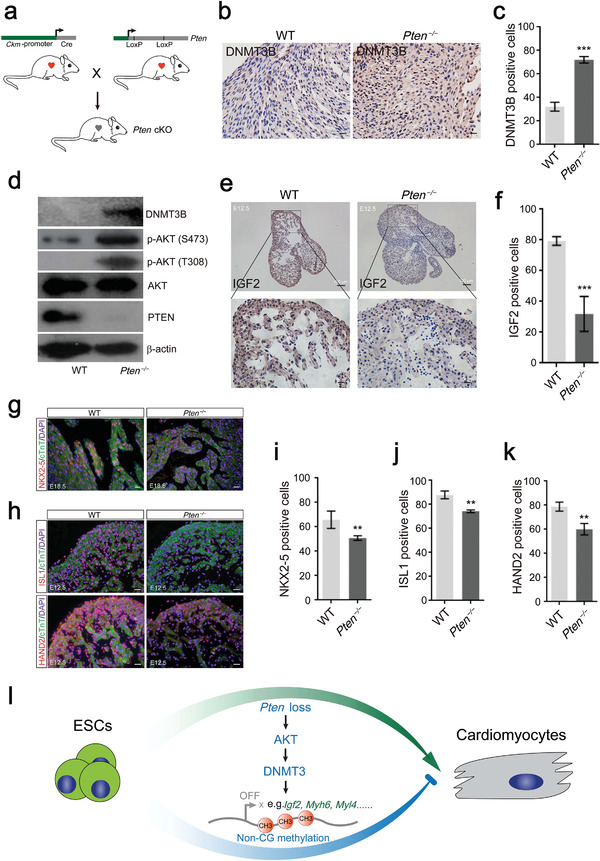
*Pten* loss suppressed cardiac development in vivo. a) A model for the generation of *Pten* conditional knockout mice. b) Immunohistochemistry experiment showing the expression of DNMT3B in the hearts of WT and *Pten^−/−^* mice. Scale bars, 20 µm. c) Quantification of the DNMT3B‐positive cells in WT and *Pten^−/−^* hearts. Error bars indicate the mean ± SD (*n* = 5). *p* values were calculated by the Student's *t*‐test: ***p < 0.001. d) The expression pattern of PTEN/AKT/DNMT3B signaling proteins in WT and *Pten^−/−^* hearts. e) Immunohistochemistry experiment showing the expression of IGF2 in the hearts of WT and *Pten^−/−^* mice at E12.5. Scale bars, 100 µm and 20 µm. f) Quantification of the IGF2‐positive cells in WT and *Pten^−/−^* heart at E12.5. Error bars indicate the mean ± SD (*n* = 5). *p* values were calculated by the Student's *t*‐test: ***p < 0.001. g) Immunofluorescence experiment showing the expression of NKX2‐5 in the hearts of WT and *Pten^−/−^* mice at E18.5. Scale bars, 20 µm. h) Immunofluorescence experiment showing the expression of ISL1 and HAND2 in the hearts of WT and *Pten^−/−^* mice at E12.5. Scale bars, 50 µm. i–k) Quantification of the NKX2‐5‐positive, ISL1‐positive, and HAND2‐positive cells in WT and *Pten^−/−^* hearts. Error bars indicate the mean ± SD (*n* = 5). *p* values were calculated by the Student's *t*‐test: **p < 0.01. l) A model of how *Pten* deletion suppresses cardiomyocyte differentiation. During cardiomyocyte differentiation from ESCs, *Pten* suppresses the expression of the *Dnmt3* family; the loss of *Pten* promotes the non‐CG methylation of cardiomyocyte genes and *Igf2*, which leads to the inhibition of cardiomyocyte differentiation.

To investigate the role of PTEN in regulating cardiogenesis in vivo, floxed *Pten* mice were mated with *Ckm* promoter‐Cre mice to generate *Pten* conditional knockout (*Pten* cKO) mice (**Figure** **8**a). Consistent with the previous description about *Pten*’s role in regulating cardiac hypertrophy,^[^ ^48^ ^]^ *Pten* loss also resulted in increased heart size in our experiments (Figure S8a,b, Supporting Information). In addition, the immunohistochemical staining showed a higher expression of DNMT3B in the *Pten* cKO group compared to the WT group (Figure 8b,c), and there was no difference in the expression of Ki67 (Figure S8c,d, Supporting Information). We also verified the PTEN/AKT/DNMT3 pathway in vivo by Western blot and showed that AKT activity was increased by phosphorylation at S473 and T308 and DNMT3B was induced in the *Pten* cKO heart (Figure 8d). On the other hand, the IGF2 expression was potently suppressed in *Pten* cKO mice at E12.5 (Figure 8e,f). These results are consistent with our in vitro data that *Pten* deletion affects cardiomyocyte differentiation by modulating IGF2. NKX2‐5 is a cardiac progenitor cell marker that is expressed in the heart throughout life.^[^ ^49^ ^]^ ISL1 and HAND2‐expressing cardiac progenitors contribute to the generation of a majority of cells of the heart.^[^ ^50^ ^]^ From our results, *Pten* loss significantly suppressed the proportion of NKX2‐5‐positive cells at E18.5 and reduced the proportion of ISL1‐positive and HAND2‐positive cells at E12.5 (Figure 8g–k), which suggests that PTEN regulates cardiogenesis at different developmental stages. We also compared the non‐CG methylation of *Tpm1* and *Myl4* gene in WT and *Pten* cKO mice by bisulfite sequencing PCR. As expected, the non‐CG methylation levels were increased by *Pten* deletion (Figure S8e, Supporting Information). Taken together, our results confirm that *Pten* loss induces the expression of DNMT3 by activating AKT and suppresses cardiomyocyte differentiation through the non‐CG methylation (Figure 8l).

## 3 Discussion

Cardiomyocyte differentiation is regulated by complex signaling pathways and transcription factor networks. We presented a growth factor‐free protocol for cardiomyocyte differentiation from mouse ESCs and reveal that *Pten* regulates cardiomyocyte differentiation by modulating non‐CG DNA methylation via *Dnmt3*.

It has been reported that the PI3K/PTEN signaling pathway regulates myocardial contractility and cell size.^[^ ^11^ ^]^ In this study, we showed that *Pten* deletion downregulates the expression of cardiomyocyte markers and reduces the cardiomyocyte beating rate. Apart from a well‐known tumor suppressor function, our results indicate that PTEN is also essential for early cardiogenesis and likely plays other roles in embryonic development. PTEN negatively regulates the PI3K signaling pathway to inhibit AKT activity,^[^ ^51^ ^]^ and AKT inactivation has been shown to downregulate DNMT3B expression and to enhance the degradation of DNMT3B.^[^ ^33^ ^]^ FoxO3a, a downstream effector of AKT, was shown to negatively regulate *Dnmt3b* expression by interacting with the *Dnmt3b* promoter.^[^ ^34^ ^]^ In this study, we demonstrated the loss of *Pten* enhances AKT activity, which triggers the translocation of FoxO3a from the nucleus and leads to a derepression of the *Dnmt3b* promoter. In addition, *Dnmt3l* was also induced by *Pten^−/−^*, and the expression of *Dnmt3l* and *Dnmt3b* might not be induced by *Pten^−/−^* at the same time point (Figure 3c–g), but we still could not clarify the regulation mechanism of the induction of *Dnmt3l* by AKT. DNMT3L bind to DNMT3A and DNMT3B to stimulate de novo methylation.^[^ ^52^ ^]^ We supposed that the regulation mechanism of the induction of *Dnmt3l* and *Dnmt3b* might be different, and further investigation is needed in the future.

We performed RNA‐seq and WGBS to analyze the transcriptomes and methylomes for the cardiomyocytes derived from WT and *Pten^−/−^* ESCs (Figures 2c,d,4). To exclude the possibility that non‐cardiomyocytes would contaminate the sequencing results, we purified the induced cardiomyocytes by sorting the cTnT‐positive cells before Q‐PCR and bisulfite sequencing analyses. The mRNA level of a series of cardiomyocyte genes was downregulated, and the *Dnmt3* levels were increased in *Pten^−/−^* cells (Figure S9a–c, Supporting Information). In addition, the non‐CG methylation of *Igf2* locus and *Tpm1* and *Myl4* gene bodies was higher in *Pten^−/−^* group than WT group (Figure S9d, Supporting Information). A previous study reported that CG methylation is dynamically changed during cardiomyocyte development and disease states,^[^ ^53^ ^]^ and the global methylation profiles can reflect the different stages of cardiomyocyte development including ESC, primitive mesoderm, cardiac mesoderm, and cardiomyocyte.^[^ ^54^ ^]^ From our results, there was no significant difference in CG methylation level in the genic, upstream, or downstream regions of the whole genes of WT EBs and cardiomyocytes (Figure 4c). We supposed that this might be due to a difference in sample types and analytical perspectives.

The loss of *Pten* induces cardiomyocyte hypertrophy,^[^ ^48^ ^]^ and DNA methylation has been described to control cardiomyocyte genes in cardiac hypertrophy.^[^ ^39^ ^]^ In our study, cardiomyocytes derived from *Pten^−/−^* ESCs showed larger cell size (Figure S9e,f, Supporting Information). The rescue of deficiency in cardiomyocyte differentiation by *Dnmt3l^−/−^/Dnmt3b^−/−^* suggests the *Pten^−/−^*‐induced *Dnmt3* may be involved in the regulation of cardiomyocyte hypertrophy. We measured the cell size of cardiomyocytes derived from *Dnmt3l/Dnmt3b‐*overexpressed (*Dnmt3l/Dnmt3b‐*OE) ESCs and found that *Dnmt3l/Dnmt3b‐*OE potently promoted the cell size of cardiomyocytes (Figure S9g,h, Supporting Information). In contrast, *Dnmt3l^−/−^/Dnmt3b^−/−^* rescued the hypertrophy induced by *Pten* deletion (Figure S9i,j, Supporting Information). These results indicate that *Pten^−/−^*‐induced hypertrophy might be caused by the induction of DNMT3. We also identified DMRs in the context of mCG, mCHG, and mCHH when comparing the methylomes of WT and *Pten^−/−^* cardiomyocytes and EBs (Figure S10a,b, Supporting Information). Cardiac disease‐related signaling pathways were enriched when comparing the DMR‐related genes of WT and *Pten^−/−^* cardiomyocytes in the context of mCHG (Figure S10c, Supporting Information). These data indicate that cardiac development is regulated by DNA methylation, which is modulated by *Pten*.

IGF signaling directs ventricular cardiomyocyte proliferation and is required for the heart development.^[^ ^15^, ^47^ ^]^ The *Igf2* and *H19* genes are expressed after imprinting, and their expression levels are determined by the methylation levels of the promoter, DMR, and imprinting control regions. We found that *Pten* deletion potently suppressed cardiomyocyte differentiation by blocking the expression of *Igf2*, but the direct relationship between *Pten* and *Igf2* in regulating cardiomyocyte development was unclear. The non‐CG methylation of *Igf2* was increased in *Pten^−/−^* cardiomyocytes (Figure 6d,e). The bisulfite sequencing PCR results showed that the non‐CG methylation level of *Igf2* locus was increased by *Pten* deletion not only in cardiomyocytes but also embryoid bodies (Figure S10d,e, Supporting Information). In addition, *Dnmt3l^−/−^/Dnmt3b^−/−^* double knockout could also recover the non‐CG methylation pattern of the *Igf2* locus (Figure S10f, Supporting Information). We therefore proposed that the expression of DNMT3 induced by *Pten* deficiency might suppress *Igf2* expression through non‐CG methylation. On the other hand, the induction of DNMT3 expression also promoted the non‐CG methylation of cardiomyocyte marker genes (Figure 5; Figure S8e, Supporting Information).

It is well known that IGF1R is a receptor of IGF2. In our study, the cardiomyocyte differentiation was suppressed by *Igf2* deletion or *Igf1r* deletion (Figure 7), and IGF2 supplementation in *Pten^−/−^* cells partially rescued the expression of cardiomyocyte markers (Figure S7b, Supporting Information). These results indicate that *Igf2* regulates cardiomyocyte differentiation through *Igf1r*. IGF2 triggers both the AKT and ERK1/2 signaling pathways.^[^ ^55^ ^]^ The dynamic AKT activities in the WT and *Igf2^−/−^* cells at different time points during cardiomyocyte differentiation showed that there was nearly no difference at the initial stages (Figure S7c, Supporting Information), while AKT activity was continually activated by *Pten* deletion (Figure S7d, Supporting Information). We proposed that it might be due to the low expression of IGF2 at the early stages, and the low expression of IGF2 could not trigger AKT. In addition, *Pten* loss might contribute more to the AKT activity and counteract the effect of decreased *Igf2* on AKT activity. Thus, the AKT activity was still activated in the *Pten* deficient group, even though the *Igf2* expression was decreased (Figures 3h and 6a). This suggested that IGF2/IGF1R might promote cardiomyocytes differentiation through other signaling pathways than PI3K/AKT pathway. MEK1 and ERK1/2 are key regulators of cardiac hypertrophy and myocyte survival in response to many different stress stimuli.^[^ ^46^, ^56^ ^]^ *Igf2* or *Igf1r* deletion reduces ERK activity, and the presence of IGF2 in culture medium was found to promote the phosphorylation of ERK1/2 (Figure 7i–k). These results suggested that IGF2 might induce cardiomyocyte differentiation through the MAPK/ERK signaling pathway.

IGF2 is also known to play a role in cell proliferation. To examine whether IGF2/IGF1R mediates cardiomyocyte differentiation and proliferation sequentially, we assessed the proliferation ability of WT and *Igf2^−/−^* ESCs, and found that *Igf2^−/−^* suppresses the cell proliferation (Figure S11a, Supporting Information). We also compared the transcriptome of WT and *Igf2^−/−^* EBs on day 6 of cardiomyocyte differentiation. The expression of a series of proliferation‐related genes was downregulated by *Igf2^−/−^* (Figure S11b, Supporting Information). Furthermore, we examined the proportion of Ki67‐positive cells for the WT and *Igf2^−/−^* cardiomyocytes. Our results showed that *Igf2* deficiency reduced the proportion of Ki67‐positive cells, while *Igf1r* deletion has no effects (Figure S11c, Supporting Information). These results indicate that other signals may be involved in cell proliferation. IGF2/INSR is a well‐known signaling pathway involved in the regulation of cell proliferation. We generated *Insr^−/−^* ESCs by the CRISPR‐Cas9 system and assessed the cell proliferation ability. Intriguingly, the proliferation of both ESCs and cardiomyocytes was inhibited by *Insr* deletion (Figure S11d–f, Supporting Information), but there seems no difference in the cardiomyocyte differentiation between WT and *Insr^−/−^* groups (Figure S11g,h, Supporting Information). We supposed that IGF2/IGF1R was involved in cardiomyocyte differentiation and IGF2/INSR was involved in cell proliferation.

We utilized *Ckm* promoter‐Cre mice to generate *Pten* cKO mice and verified the relationship of DNA methylation and cardiac development. Besides cardiomyocytes, *Ckm* is also expressed in skeletal muscle cells. The loss of *Pten* in all cells results in early embryonic lethality,^[^ ^57^ ^]^ suggesting that *Pten* regulates various biological processes in different cell types. We revealed that *Pten* loss suppressed cardiac development via non‐CG DNA methylation, which was seldomly reported in the mammalian system. The WGBS showed that the non‐CG methylation levels of large number of genes were upregulated by *Pten* loss (Figure 4f; Figure S6f, Supporting Information). Thus, we postulated that the loss of *Pten* might affect other biological processes in various cell types, including skeletal muscle cells, via non‐CG DNA methylation, which awaits further investigations in the future.

## 4 Conclusions

Collectively, we reported that *Pten* deletion suppresses cardiomyocyte differentiation by promoting non‐CG DNA methylation. *Pten* loss significantly induces the expression of DNMT3B and DNMT3L by activating AKT and suppresses the expression of *Igf2* which was involved in regulating cardiomyocyte differentiation. We also verified the role of PTEN/AKT/DNMT3 pathway in cardiomyocyte differentiation in vivo.

## 5 Experimental Section

### Cell Line and Mice

The mouse E14 cell line was purchased from the ATCC (ES‐D3, ATCC, CRL‐1934). Mouse ESCs were cultured as previously described.^[^ ^29^ ^]^ Briefly, mouse ESCs were cultured in DMEM/F12 containing GlutaMAX and sodium pyruvate (Life Technologies, 10565) with 15% fetal bovine serum (Hyclone, SH30071), non‐essential amino acids solution (Life Technologies, 11140), HEPES (Life Technologies, 15630080), *β*‐mercaptoethanol (Life Technologies, 31350) with 1 × 10^3^ units/ml LIF (Millipore, ESG1106), 1 × 10^−6^ m PD0325901 (Sigma, PZ0162), and 2.5 × 10^−6^ m CHIR99021 (Sigma, SML1046). The mouse ESCs were grown on gelatin‐coated plates and the medium was replaced every other day. To generate conditional knockout mice, exon 2 of the *Pten* gene was flanked with LoxP sites, then *Pten* ^flox/flox^ mice were crossed with *Ckm*‐Cre transgenic mice to generate conditional knockout mice. The primers used for genotyping and the sequences are shown in Table S1, Supporting Information. Mouse procedures were performed in accordance with the recommendations of the Animal Experimentation Ethics Committee of The Chinese University of Hong Kong. The protocol was approved by the Animal Experimentation Ethics Committee of The Chinese University of Hong Kong (Ref No. 19/129/MIS‐4‐B).

### Cardiomyocyte Differentiation

ESCs were cultured in ESC culture medium without 2iL (2i, PD0325901 and CHIR99021; L, LIF) to form EBs. Briefly, EBs were formed using the hanging drop method with 250 cells/drop in DMEM/F12 media containing GlutaMAX and sodium pyruvate with 15% fetal bovine serum, non‐essential amino acids solution, HEPES, and *β*‐mercaptoethanol for 5 days. About 70 EBs were collected and transferred to a 35‐mm petri dish with CMCM containing DMEM/F12 (Life Technologies, 10565), non‐essential amino acids solution, heparin (Sigma, H3149), and N2 (Life Technologies, 17502). After 2 days of suspension culture in CMCM, EBs were collected and transferred to gelatin‐coated tissue culture plate with CMCM. After about 9 days, beating cardiomyocytes were generated.

### Igf2, Dnmt3l, Dnmt3b, Igf1r, Insr, and Foxo3 Deletion in Mouse ESCs

*Pten^−/−^* ESCs were generated in the authors' previous work,^[^ ^29^ ^]^ and the Cas9 target sequences for *Igf2, Dnmt3l, Dnmt3b, Igf1r, Insr, and Foxo3* deletion were designed as previously detailed.^[^ ^58^ ^]^ Guide sequences of the sgRNA were incorporated into the pSpCas9(BB)‐2A‐GFP vector (Addgene, 48138) containing the *Cas9* and green fluorescent protein (*GFP*) genes. ESCs were cultured in 6‐well plates and transfected with constructs containing *Cas9* and target sequences using Lipofectamine 3000 (Invitrogen); two days later, the cells were subjected to sorting of GFP‐positive cells. The enriched gene‐modified cell populations were cultured in 60‐mm dishes for 4–5 days, and each cell clone was passaged into 24‐well plates. Finally, the *Igf2^−/−^*, *Igf1r^−/−^*, *Pten^−/−^*/*Dnmt3l^−/−^*, *Pten^−/−^*/*Dnmt3l^−/−^*/*Dnmt3b^−/−^*, *Insr^−/−^*, and *Foxo3^−/−^* cell lines were identified by DNA sequencing.

### Western Blot

Cells were scraped from culture plates and incubated for 20 min in ice‐cold lysis buffer containing protease inhibitor cocktails. Nuclear and cytoplasmic fractions were prepared as described previously.^[^ ^29^ ^]^ Total protein (10 µg) was separated by SDS‐PAGE and transferred to a PVDF membrane (Bio‐Rad). The membrane was incubated with primary antibodies, and the protein was visualized with ECL (HRP) (Millipore). The following antibodies were used for western blot analysis: anti‐Pten (Cell Signaling Technology, 9188), anti‐*β*‐actin (Immunoway, YM3028), anti‐p‐T308‐Akt (Cell Signaling Technology, 13038), anti‐p‐S473‐Akt (Cell Signaling Technology, 4060), anti‐Akt (Cell Signaling Technology, 4691), anti‐cTnT (Abcam, ab8295), anti‐Dnmt3l (Cell Signaling Technology, 13451), anti‐Dnmt3b (Cell Signaling Technology, 48488), anti‐Dnmt3b (ab122932, Abcam), anti‐Igf1r (Cell Signaling Technology, 3027), anti‐p‐S253‐FoxO3a (Abcam, ab47285), anti‐p‐T32‐FoxO3a (Cell Signaling Technology, 9464), anti‐FoxO3a (Cell Signaling Technology, 2497), anti‐Phospho‐p44/42 MAPK (Erk1/2) (Cell Signaling Technology, 4370), anti‐ Insulin Receptor *β* (Abcam, ab69508), anti‐p44/42 MAPK (Erk1/2) (Cell Signaling Technology, 4695), anti‐*β*‐Tubulin (Cell Signaling Technology, 2146), and anti‐Lamin A/C (Cell Signaling Technology, 4777).

### Bisulfite Sequencing PCR

Bisulfite sequencing PCR was performed to analyze the methylation of CG, CHG, and CHH using the EZ DNA Methylation‐Gold kit (Zymo Research, D5005) according to the manufacture's instruction. Briefly, DNA from WT and *Pten^−/−^* EBs and cardiomyocytes was treated with sodium bisulfite to convert unmethylated cytosine into uracil. The converted DNA was used as the template for PCR using the primers listed in Table S1, Supporting Information, and the PCR product was inserted into the pMD18‐T vector (Takara) and sequenced by Sanger sequencing.

### WGBS

For WGBS library construction, genomic DNA was fragmented by sonication to an average size of approximately 250 bp, and DNA was bisulfite converted using the EZ DNA Methylation‐Gold kit. Different insert size fragments were purified and amplified by PCR. Finally, sequencing was performed using the DNBseq platforms (BGI Genomics).

### Methylation Analysis

The raw data were filtered, and the clean data were mapped to the reference genome (Mouse GRCm38/mm10). The uniquely mapped data provided cytosine methylation information throughout the whole genome, and this information was used for standard bioinformatics analysis.

### Methylation Level Determination

The methylation level was determined by dividing the number of reads covering each mC by the total number of covering that cytosine, which was also equal to the mC/C ratio at each reference cytosine according to the formula Rm_average_ = Nm_all_ × 100% / (Nm_all_ + Nnm_all_), where Nm represents the number of mC reads and Nnm represents the number of non‐methylation reads.

### RNA‐seq

RNA‐seq for WT and *Pten^−/−^* cardiomyocytes and WT and *Igf2^−/−^* EBs were performed. Total RNA was isolated from the cells according to the manufacturer's instructions using TRIzol reagent, and the RNA was converted into a template molecules library for sequencing on a BGISEQ‐500 (BGI, ShenZhen, China).

### Statistical Analysis

At least three independent sets of experiments for each condition were performed in triplicate. All statistical analyses were conducted using GraphPad Prism (version 6). All data were presented in mean ± SD. Independent biological replicates were used to determine *n* values. Statistical significance was calculated by a Student's *t*‐test between the indicated groups, and statistical significance threshold of each test was set at *p* < 0.05: ns = not significant, *p* > 0.05; *p < 0.05; **p < 0.01; and ***p < 0.001.

## Conflict of Interest

The authors declare no conflict of interest.

## Supporting information

Supporting Information

Supplemental Video 1

Supplemental Video 2

Supplemental Video 3

Supplemental Video 4

Supplemental Video 5

Supplemental Table 1

Supplemental Table 2

Supplemental Table 3

Supplemental Table 4

Supplemental Table 5

Supplemental Table 6

Supplemental Table 7

Supplemental Table 8

Supplemental Table 9

Supplemental Table 10

Supplemental Table 11

Supplemental Table 12

## Data Availability

The RNA‐seq raw data and normalized mapped reads are available from the Gene Expression Omnibus (GEO) under accession numbers GSE117280, GSE173810, and GSE156907. The WGBS raw datasets have been deposited in GEO under accession number GSE160236. All other data supporting the findings of this study are available from the corresponding author upon reasonable request.
