# Supplementary material for: Pten Regulates Cardiomyocyte Differentiation by Modulating Non‐CG Methylation via Dnmt3
Source: Adv Sci (Weinh). 2021 Jul 11;8(17):2100849. doi: 10.1002/advs.202100849 (PMC8425920; doi:10.1002/advs.202100849)
Supplement: Supplementary file 1 — Supporting Information [file ADVS-8-2100849-s010.pdf]

© 2021 Wiley-VCH GmbH

ADVANCED SCIENCE
Open Access

Supporting Information

for *Adv. Sci.,* DOI: 10.1002/advs.202100849

Pten Regulates Cardiomyocyte Differentiation by Modulating Non-CG methylation via *Dnmt3*

Wuming Wang, Gang Lu, Hong-Bin Liu, Zhiqiang Xiong, Ho-Duen Leung, Ruican Cao, Alan Lap-Yin Pang, Xianwei Su, Patrick Wai Nok Law, Zhiju Zhao, Zi-Jiang Chen, Wai-Yee Chan**

Supporting Information

Pten Regulates Cardiomyocyte Differentiation by Modulating Non-CG methylation via *Dnmt3*

Wuming Wang, Gang Lu, Hong-Bin Liu, Zhiqiang Xiong, Ho-Duen Leung, Ruican Cao, Alan Lap-Yin Pang, Xianwei Su, Patrick Wai Nok Law, Zhiju Zhao, Zi-Jiang Chen, Wai-Yee Chan**

Supplementary Figure Legends

Video S1. The cardiomyocytes derived from ESCs can spontaneously beat.

Video S2. WT EBs show a high spontaneous beating rate during cardiomyocyte differentiation.

Video S3. *Pten*$^{-/-}$ EBs show a low spontaneous beating rate during cardiomyocyte differentiation.

Video S4. Cardiomyocytes derived from WT ESCs show spontaneous beating.

Video S5. Cells derived from *Pten*$^{-/-}$ ESCs tend to be neural stem cell-like cells.

Figure S1. Immunofluorescence of cTnT, PLN, α-Actinin, and MLC2v showed the sarcomeric striations of cardiomyocyte markers. Scale bars, 20 μm.

Figure S2. RNA-seq analysis of WT and *Pten*$^{-/-}$ cardiomyocytes. **a**) KEGG-DEG relationship network. The red and blue dots represent the up-regulated and down-regulated genes, respectively. Purple balls represent the top nine enriched pathways. **b**) Pathway functional enrichment of DEGs. **c**) Pathway functional enrichment result for up/down-regulated genes.

Figure S3. PTEN re-expression could rescue cardiomyocyte differentiation. **a**) Western blot showing the expression of PTEN after *Pten* OE. **b**) Q-PCR analysis of mRNA expression of cardiomyocyte marker genes in WT, *Pten*$^{-/-}$, and *Pten*-OE cardiomyocytes. Error bars indicate the mean ± SD (n = 3). p Values were calculated by the Student's t-test: *$p < 0.05$, **$p < 0.01$, and ***$p < 0.001$. **c**) Representative flow cytometry analysis of the proportion of cTnT-positive cells in WT, *Pten*$^{-/-}$, and *Pten*-OE cardiomyocytes. d) Q-PCR analysis of mRNA expression of cardiomyocyte marker genes in WT and *Pten*-A3 mutant

cardiomyocytes. Error bars indicate the mean ± SD (n = 3). p Values were calculated by the Student's t-test: $*p < 0.05$, $**p < 0.01$, and $***p < 0.001$. e) Dynamic expression of PTEN at different time points during cardiomyocyte differentiation from day 0 to day 11.

Figure S4. AKT-dependent phosphorylation of FoxO3a regulates the expression of *Dnmt3b*. **a**) Protein levels of DNMT3B and DNMT3L and phosphorylation levels of AKT at T308 and S473 in *Pten*$^{-/-}$ cardiomyocytes treated with DMSO and PI3K-inhibitor PX-866. **b**) Protein levels of DNMT3B and DNMT3L and phosphorylation levels of AKT at T308 and S473 in WT and PTEN-A3 mutant cardiomyocytes. **c**) Protein levels of FoxO3a in the cytoplasm and nucleus of DMSO-treated and doxorubicin-treated cardiomyocytes. **d**) Protein level of DNMT3B in the DMSO-treated and doxorubicin-treated cardiomyocytes.

Figure S5. *Dnmt3b*$^{-/-}$/*Dnmt3l*$^{-/-}$ rescues cardiomyocyte differentiation. **a**) The protein level of DNMT3L in *Pten*$^{-/-}$ and *Pten*$^{-/-}$/*Dnmt3l*$^{-/-}$ cardiomyocytes. **b**) Q-PCR analysis of the mRNA expression of cardiomyocyte marker genes in *Pten*$^{-/-}$ and *Pten*$^{-/-}$/*Dnmt3l*$^{-/-}$ cardiomyocytes. Error bars indicate the mean ± SD (n = 3). p Values were calculated by the Student's t-test: $***p < 0.001$. **c**) Representative flow cytometry analysis of the proportion of cTnT-positive cells in *Pten*$^{-/-}$ and *Pten*$^{-/-}$/*Dnmt3l*$^{-/-}$ cardiomyocytes. **d**) The protein level of DNMT3B in *Pten*$^{-/-}$ and *Pten*$^{-/-}$/*Dnmt3l*$^{-/-}$/*Dnmt3b*$^{-/-}$ cardiomyocytes. **e**) Phase-contrast images of WT, *Pten*$^{-/-}$, and *Pten*$^{-/-}$/*Dnmt3l*$^{-/-}$/*Dnmt3b*$^{-/-}$ cardiomyocytes, and the cardiomyocytes were indicated with red arrows. Scale bars, 100 μm. **f**) Representative flow cytometry analysis of the proportion of cTnT-positive cells in WT, *Pten*$^{-/-}$, and *Pten*$^{-/-}$/*Dnmt3l*$^{-/-}$/*Dnmt3b*$^{-/-}$ cardiomyocytes. **g**) Q-PCR analysis of the mRNA expression of cardiomyocyte marker genes in WT, *Pten*$^{-/-}$, and *Pten*$^{-/-}$/*Dnmt3l*$^{-/-}$/*Dnmt3b*$^{-/-}$ cardiomyocytes. Error bars indicate the mean ± SD (n = 3). p Values were calculated by the Student's t-test: $*p < 0.05$, $**p < 0.01$, and $***p < 0.001$. **h**) Protein level of DNMT3B and DNMT3L in control and *Dnmt3b/Dnmt3l*-OE groups. **i**) Representative flow cytometry analysis of the proportion of cTnT-positive cells in control and *Dnmt3b/Dnmt3l*-OE cardiomyocytes. **j**) Q-PCR analysis of the mRNA expression of cardiomyocyte marker genes in control and *Dnmt3b/Dnmt3l*-OE cardiomyocytes. Error bars indicate the mean ± SD (n = 3). p Values were calculated by the Student's t-test: ns, not significant, $**p < 0.01$, and $***p < 0.001$.

Figure S6. Non-CG methylation in WT and *Pten*$^{-/-}$ EBs and cardiomyocytes. **a**) Distribution of the methylation level in the context of mC, mCG, mCHG, and mCHH in WT and *Pten*$^{-/-}$ cardiomyocytes. **b**) Model of cardiomyocyte differentiation from ESCs via EBs. **c**) Sequence logos are shown for bases proximal to hypermethylated CHGs in WT EBs and cardiomyocytes. **d**) Sequence logos are shown for bases proximal to hypermethylated CHHs

in WT EBs and cardiomyocytes. **e**) Correlation plot of mCHH level and cardiomyocyte gene expression. **f**) Heatmap showing the mRNA and mCHG levels of differentially expressed genes of WT and *Pten*$^{-/-}$ cardiomyocytes. See also Table S5 and S9. **g**) Heat map of CHG methylation of cardiomyocyte genes, cardiac transcription factors, imprinted genes, and pluripotency genes in *Pten*$^{-/-}$ cardiomyocytes compared to WT cardiomyocytes. See also Table S10. **h**, **i**, **j**) The genomic distribution of CHG methylation in cardiac genes, cardiac transcription factors, and imprinted genes in WT and *Pten*$^{-/-}$ cardiomyocytes.

Figure S7. IGF2/IGF1R signaling regulates cardiomyocyte differentiation. **a**) The protein level of IGF2 was measured in WT and *Pten*$^{-/-}$ cells at different time points by ELISA. Error bars indicate the mean ± SD (n = 3). **b**) Q-PCR analysis of mRNA expression of cardiomyocyte marker genes in *Pten*$^{-/-}$ cardiomyocytes treated with BSA and IGF2. Error bars indicate the mean ± SD (n = 3). *p* Values were calculated by the Student's *t*-test: ns, not significant, ***$p < 0.001$. **c**) AKT activity in the WT and *Igf2*$^{-/-}$ groups at different time points during cardiomyocyte differentiation. **d**) AKT activity in the WT and *Pten*$^{-/-}$ groups at different time points during cardiomyocyte differentiation.

Figure S8. a) Hearts of WT and *Pten* cKO mice. **b**) Weights of the hearts from WT and *Pten* cKO mice. Error bars indicate the mean ± SD (n = 4). *p* Value were calculated by the Student's *t*-test: **$p < 0.01$. **c**) Immunohistochemical staining showed the expression of Ki67 in the hearts of WT^{-} and *Pten*$^{-/-}$ mouse. Scale bars, 20 μm. **d**) Quantification for the Ki67-positive cells in WT and *Pten*$^{-/-}$ heart. Error bars indicate the mean ± SD (n = 5). ns, not significant. **e**) Non-CG methylation of the *Tpm1* and *Myl4* gene in WT and *Pten* cKO hearts by bisulfite sequencing PCR. Error bars indicate the mean ± SD (n = 8). *p* Values were calculated by the Student's *t*-test: *$p < 0.05$.

Figure S9. a) Heatmap showing the mRNA levels of cardiac genes in cTnT-positive cells sorted from WT and *Pten*$^{-/-}$ groups. See also Table S11. **b,c**) mRNA levels of *Dnmt3b* and *Dnmt3l* in cTnT-positive cells sorted from WT and *Pten*$^{-/-}$ groups. Error bars indicate the mean ± SD (n = 3). *p* Values were calculated by the Student's *t*-test: ***$p < 0.001$. **d**) After sorting the cTnT-positive cells, non-CG methylation of the *Igf2*, *Tpm1*, and *Myl4* genes in WT and *Pten*$^{-/-}$ cardiomyocytes by bisulfite sequencing PCR. Error bars indicate the mean ± SD (n = 7). *p* Values were calculated by the Student's *t*-test: *$p < 0.05$. **e**) Cardiomyocyte morphology of WT and *Pten*$^{-/-}$ cardiomyocytes. Scale bars, 20 μm. **f**) Morphometric quantification of cell surface area of WT and *Pten*$^{-/-}$ cardiomyocytes. Error bars indicate the mean ± SD (n = 15). *p* Value were calculated by the Student's *t*-test: **$p < 0.01$. **g**) Morphology of cell surface area of control and *Dnmt3l/Dnmt3b*-OE cardiomyocytes. Scale bars, 20 μm. **h**) Morphometric quantification of cell surface area of control and

Dnmt3l/Dnmt3b-OE cardiomyocytes. Error bars indicate the mean ± SD (n = 15). *p* Value were calculated by the Student's *t*-test: $^{**}p < 0.01$. **i**) Morphology of cell surface area of WT and $Pten^{-/-}/Dnmt3l^{-/-}/Dnmt3b^{-/-}$ cardiomyocytes. Scale bars, 20 μm. **j**) Morphometric quantification of cell surface area of WT and $Pten^{-/-}/Dnmt3l^{-/-}/Dnmt3b^{-/-}$ cardiomyocytes. Error bars indicate the mean ± SD (n = 15). *p* Value were calculated by the Student's *t*-test: ns, not significant.

Figure S10. **a**, **b**) Histograms of DMR size distributions between WT and $Pten^{-/-}$ cardiomyocytes and EBs (bin size 250 bp). **c**) Pathway analysis of DMR-related genes. **d**) Promoter and ICR methylation of the *Igf2* gene in WT and $Pten^{-/-}$ cardiomyocytes by bisulfite sequencing PCR. Error bars indicate the mean ± SD (n = 8). *p* Values were calculated by the Student's *t*-test: $^{*}p < 0.05$. **e**) Promoter and ICR methylation of the *Igf2* gene in WT and $Pten^{-/-}$ EBs by bisulfite sequencing PCR. Error bars indicate the mean ± SD (n = 7). *p* Values were calculated by the Student's *t*-test: $^{*}p < 0.05$. **f**) Promoter and ICR methylation of the *Igf2* locus in WT, $Pten^{-/-}$, and $Pten^{-/-}/Dnmt3l^{-/-}/Dnmt3b^{-/-}$ cardiomyocytes by bisulfite sequencing PCR. Error bars indicate the mean ± SD (n = 8). *p* Values were calculated by the Student's *t*-test: ns, not significant, $^{*}p < 0.05$.

Figure S11. IGF2/INSR signaling regulates cell proliferation. **a**) Cell proliferation ability of WT and $Igf2^{-/-}$ ESCs. Error bars indicate the mean ± SD (n = 3). *p* Values were calculated by the Student's *t*-test: $^{*}p < 0.05$ and $^{**}p < 0.01$. **b**) Heatmap showing the mRNA levels of a series of cell proliferation positive regulated genes. See also Table S12. **c**) Flow cytometry showing the proportion of Ki67-positive cells in WT, $Igf2^{-/-}$, and $Igf1r^{-/-}$ cardiomyocytes. **d**) Protein level of INSR in WT and $Insr^{-/-}$ cells. **e**) Flow cytometry showing the proportion of Ki67-positive cells in WT and $Insr^{-/-}$ cardiomyocytes. **f**) Cell proliferation ability of WT and $Insr^{-/-}$ ESCs. Error bars indicate the mean ± SD (n = 3). *p* Values were calculated by the Student's *t*-test: $^{*}p < 0.05$. **g**) Flow cytometry showing the proportion of cTnT-positive cells in WT and $Insr^{-/-}$ cardiomyocytes. **h**) Q-PCR analysis of the mRNA expression of cardiomyocyte marker genes in WT and $Insr^{-/-}$ cardiomyocytes. Error bars indicate the mean ± SD (n = 3).

Supplemental Experimental Section

Plasmid Construction: mouse *Pten*, *Dnmt3l*, and *Dnmt3b* cDNA were amplified by RT-PCR from total RNA isolated from mouse ESCs and cloned into the pCDH-EF1-MCS-BGH-PGK-copGFP-T2A-Puro Vector (System Biosciences, CD550A-1).

Immunofluorescence: cardiomyocytes derived from ESCs were fixed in 4% PFA for 20 min and incubated with 0.3% Triton-X 100 for 10 min. After blocking with 5% bovine serum albumin in PBS, the cells were stained with primary antibodies and incubated overnight at 4 °C. The next day, samples were incubated with donkey anti-rabbit IgG highly cross-adsorbed secondary antibody (Life Technologies, A16036) and donkey anti-mouse IgG highly cross-adsorbed secondary antibody (Life Technologies, A16018) for 2 h at room temperature. The following antibodies were used for immunofluorescence analysis: anti-phospholamban (ab2865), anti-cTnT (Abcam, ab8295), anti-MLC2v (Protein Tech Group, PTG10906-1-AP), and anti-α-Actinin (Sigma, A7811).

Histological Analysis: Mouse hearts were fixed overnight in 4% PFA, dehydrated, paraffin-embedded, and sectioned at a thickness of 5 μm. For immunohistochemistry, the sections were blocked for 30 mins at room temperature and incubated with primary antibodies overnight at 4 °C. The next day, samples were incubated with secondary antibody (Abcam, #ab64261) for 1 h, and one drop of DAB substrate was added to the sample (Abcam, #ab64261). The following primary antibody was used for immunohistochemistry and immunofluorescence: anti-Dnmt3l (Abcam, ab194094), anti-Dnmt3b (Abcam, ab2851), anti-IGF2 (Abcam, ab262713), anti-Ki-67 (Cell Signaling Technology, 9129), anti-ISL1 (Abclonal, A0871), anti-HAND2 (Abclonal, A7044), and NKX2-5 (Abcam, ab272914).

Real-time PCR Analysis: The cells were collected, and total RNA was isolated using Trizol reagent (Invitrogen). About 400 ng of RNA was reverse-transcribed, and mRNA levels were measured by SYBR Green quantitative PCR using the $^{\Delta\Delta}$Ct method. We chose *Gapdh* as the reference gene for normalization, and technical replicates were carried out for all quantitative PCRs. The primer sequences are listed in Table S1.

Flow Cytometry: The cells were fixed in 4% PFA for 15 min, permeabilized by 0.3% Triton-X 100, and stained with anti-cTnT antibody (Abcam, ab8295) and anti-Ki67 (Abcam, ab16667) and subsequently probed by a goat anti-mouse IgG conjugated with Alexa Fluor 647 (Abcam, ab150115).

Purification of cTnT-positive cells: The cells derived from WT and *Pten*$^{-/-}$ ESCs were stained with anti-cTnT antibody (Abcam, ab8295) and subsequently probed by a goat anti-mouse IgG conjugated with Alexa Fluor 647 (Abcam, ab150115). cTnT-positive cells were sorted by performing flow cytometry. Total RNA was extracted from about 2×10^5 cTnT-positive cells from each group for by Q-PCR. Genomic DNA was extracted from about 2×10^5 cTnT-

positive cells from each group for bisulfite sequencing to examine the methylation level of WT and *Pten*$^{-/-}$ cardiomyocytes.

ChIP-qPCR: ChIP was performed using the Pierce Magnetic CHIP Kit (26157, Thermo Scientific) according to the manufacturer's instructions. Briefly, 4×10^6 cells were cross-linked with 1% formaldehyde for 10 min and incubated with glycine for 5 min at room temperature. The crosslinked cells were lysed with membrane extraction buffer, and nuclei were digested with MNase. Subsequently, the lysates were sonicated. An aliquot of 10% was used as the input control. The sonicated solution was diluted and incubated with antibody against DNMT3B (ab122932, Abcam). The IP reactions were incubated with Protein A/G magnetic beads. Following elution, protein-DNA complexes were reverse-crosslinked by heating at 65 °C, and the DNA was purified with column. qPCR was then carried out using SYBR Green chemistry, and the data were presented as fold enrichment over the input control (10%).

Luciferase Reporter Assays: DNA fragments of the *Tpm1* and *Myl4* promoters from WT and *Pten*$^{-/-}$ cardiomyocytes were inserted into the pGL3-Basic reporter vector (Promega) to generate reporter constructs. 239T cells were transiently transfected with respective reporter construct; a *Renilla* luciferase reporter vector was co-transfected as a normalization control. Firefly and *Renilla* luciferase activities were measured using the Dual Glo reagent.

DMR Detection: Putative DMRs were identified by comparison of the sample1 and sample 2 methylomes using windows that contained at least 5 CG (CHG or CHH) sites with a 2-fold change in methylation level and a Fisher test value of ≤0.05. Two nearby DMRs were considered independent and joined into one continuous DMR if the genomic region from the start of an upstream DMR to the end of a downstream DMR also had 2-fold methylation level differences between sample 1 and sample 2 with a *p*-value of ≤0.05.

Analysis of the DNA methylation level within genic regions: We analyzed the methylation level of upstream, first exon, first intron, internal exon, internal intron, last exon, and downstream regions of the differentially expressed genes, and exon regions were split to 10 windows, while non-exon regions were split to 20 windows. We calculated the methylation level of windows for each gene with the average value (number of methylation reads / number of total reads) of the methylated points that the number of covered reads is above 4 and the copy number is less than 2000 in the window.

Integrative Genomics Viewer (IGV): For each sample (WT EBs, *Pten*$^{-/-}$ EBs, WT cardiomyocytes, and *Pten*$^{-/-}$ cardiomyocytes), the positions of interest in specific chromosomes' count files were extracted using awk in the command line. Bedgraph files for each methylation pattern (CG/CHG/CHH) were generated from the extracted files using grep in the command line. The bedgraph files were then input into IGV v2.8.0 for visualization. Samples with the same extracted regions and methylation information were visualized together at the same time.

ELISA: The levels of IGF2 were determined by the Mouse IGF2 ELISA Kit (ab100696, Abcam) following the manufacturer's guidelines.

Figure S1

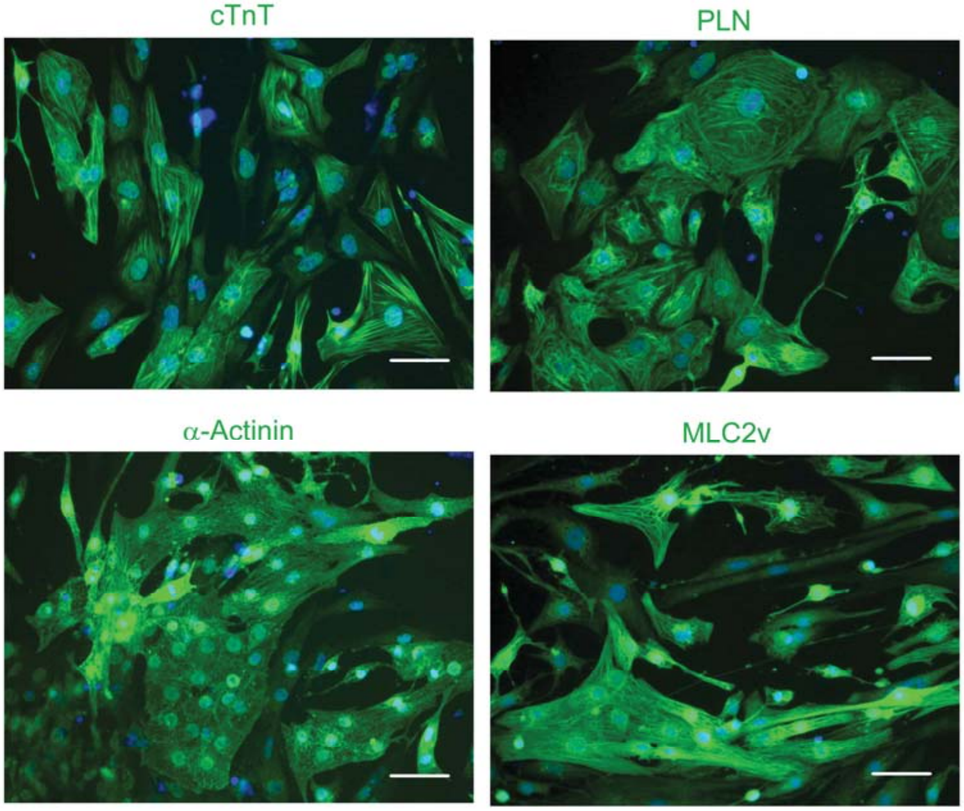

Figure S2

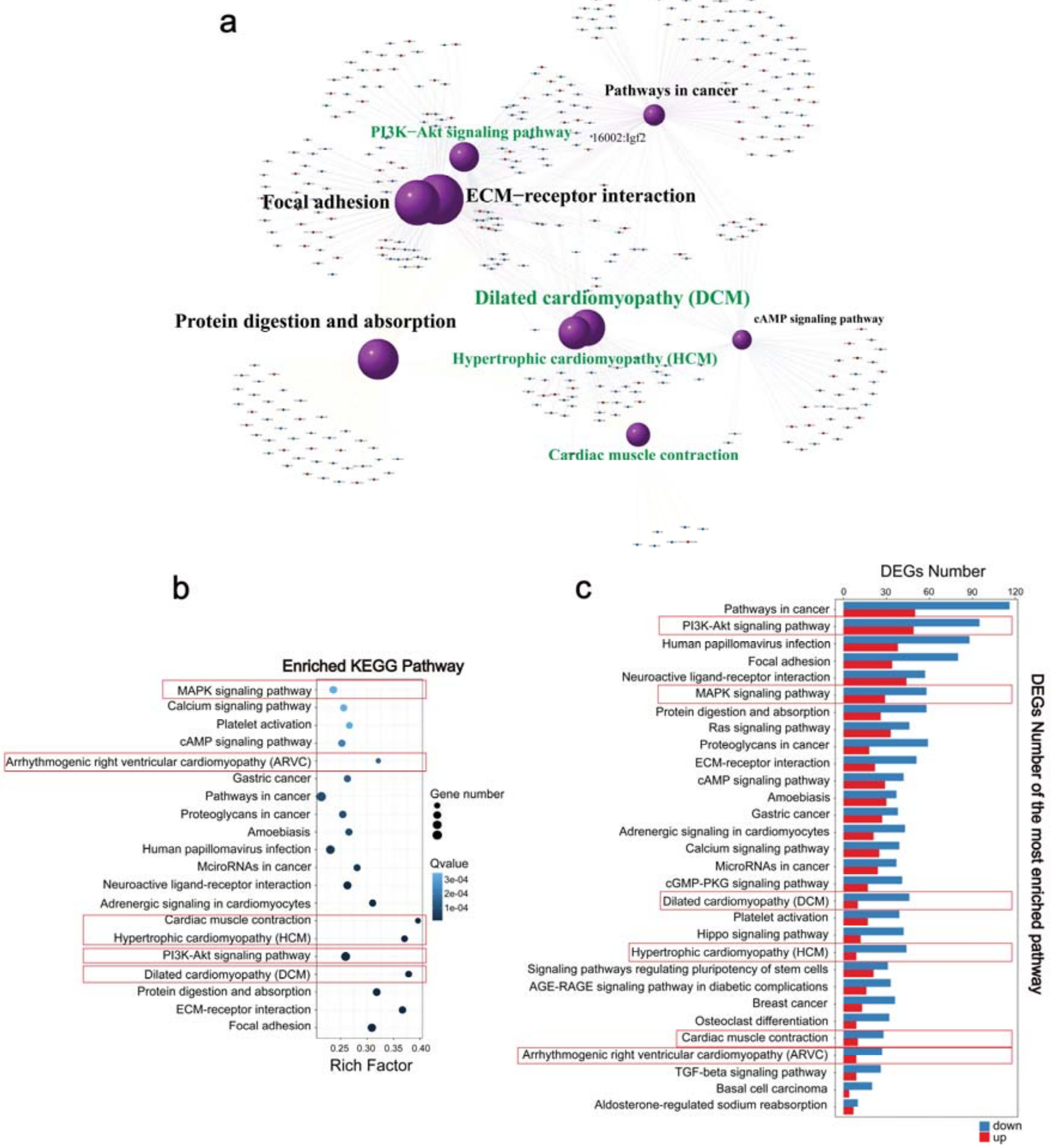
a
Pathways in cancer
PI3K−Akt signaling pathway
16002:Igf2
Focal adhesion
ECM−receptor interaction
Dilated cardiomyopathy (DCM)
Protein digestion and absorption
cAMP signaling pathway
Hypertrophic cardiomyopathy (HCM)
Cardiac muscle contraction
b
Enriched KEGG Pathway
MAPK signaling pathway
Calcium signaling pathway
Platelet activation
cAMP signaling pathway
Arrhythmogenic right ventricular cardiomyopathy (ARVC)
Gastric cancer
Pathways in cancer
Proteoglycans in cancer
Amoebiasis
Human papillomavirus infection
MciroRNAs in cancer
Neuroactive ligand-receptor interaction
Adrenergic signaling in cardiomyocytes
Cardiac muscle contraction
Hypertrophic cardiomyopathy (HCM)
PI3K-Akt signaling pathway
Dilated cardiomyopathy (DCM)
Protein digestion and absorption
ECM-receptor interaction
Focal adhesion
0.25 0.30 0.35 0.40
Rich Factor
Gene number
Qvalue
3e-04
2e-04
1e-04
c
DEGs Number
0 30 60 90 120
Pathways in cancer
PI3K-Akt signaling pathway
Human papillomavirus infection
Focal adhesion
Neuroactive ligand-receptor interaction
MAPK signaling pathway
Protein digestion and absorption
Ras signaling pathway
Proteoglycans in cancer
ECM-receptor interaction
cAMP signaling pathway
Amoebiasis
Gastric cancer
Adrenergic signaling in cardiomyocytes
Calcium signaling pathway
MicroRNAs in cancer
cGMP-PKG signaling pathway
Dilated cardiomyopathy (DCM)
Platelet activation
Hippo signaling pathway
Hypertrophic cardiomyopathy (HCM)
Signaling pathways regulating pluripotency of stem cells
AGE-RAGE signaling pathway in diabetic complications
Breast cancer
Osteoclast differentiation
Cardiac muscle contraction
Arrhythmogenic right ventricular cardiomyopathy (ARVC)
TGF-beta signaling pathway
Basal cell carcinoma
Aldosterone-regulated sodium reabsorption
down
up
DEGs Number of the most enriched pathway

Figure S3

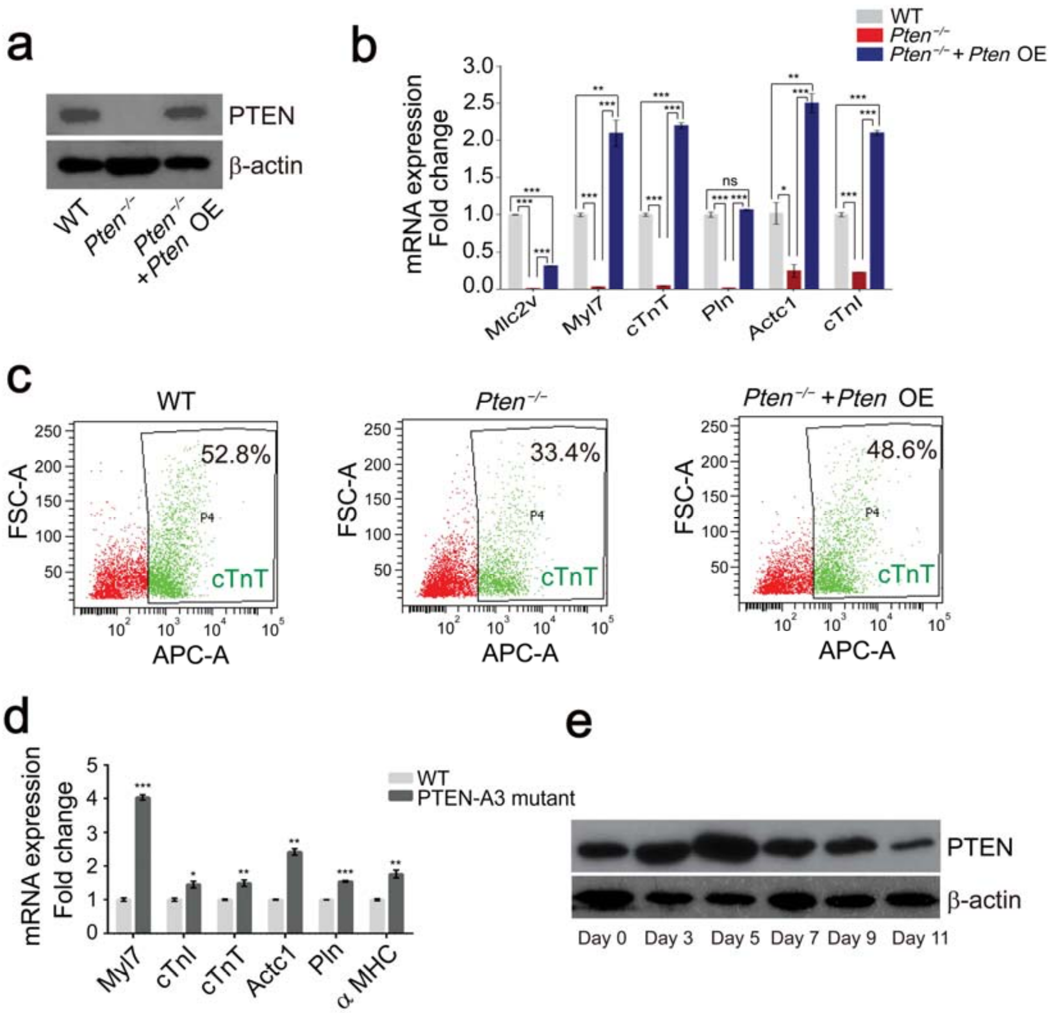
a
PTEN
β-actin
WT
Pten−/−
Pten−/− + Pten OE
b
WT
Pten−/−
Pten−/− + Pten OE
mRNA expression Fold change
Mlc2v
Myl7
cTnT
Pln
Actc1
cTnI
ns
c
WT
Pten−/−
Pten−/− + Pten OE
52.8%
33.4%
48.6%
cTnT
P4
FSC-A
APC-A
d
mRNA expression Fold change
WT
PTEN-A3 mutant
Myl7
cTnI
cTnT
Actc1
Pln
α MHC
e
PTEN
β-actin
Day 0
Day 3
Day 5
Day 7
Day 9
Day 11

Figure S4

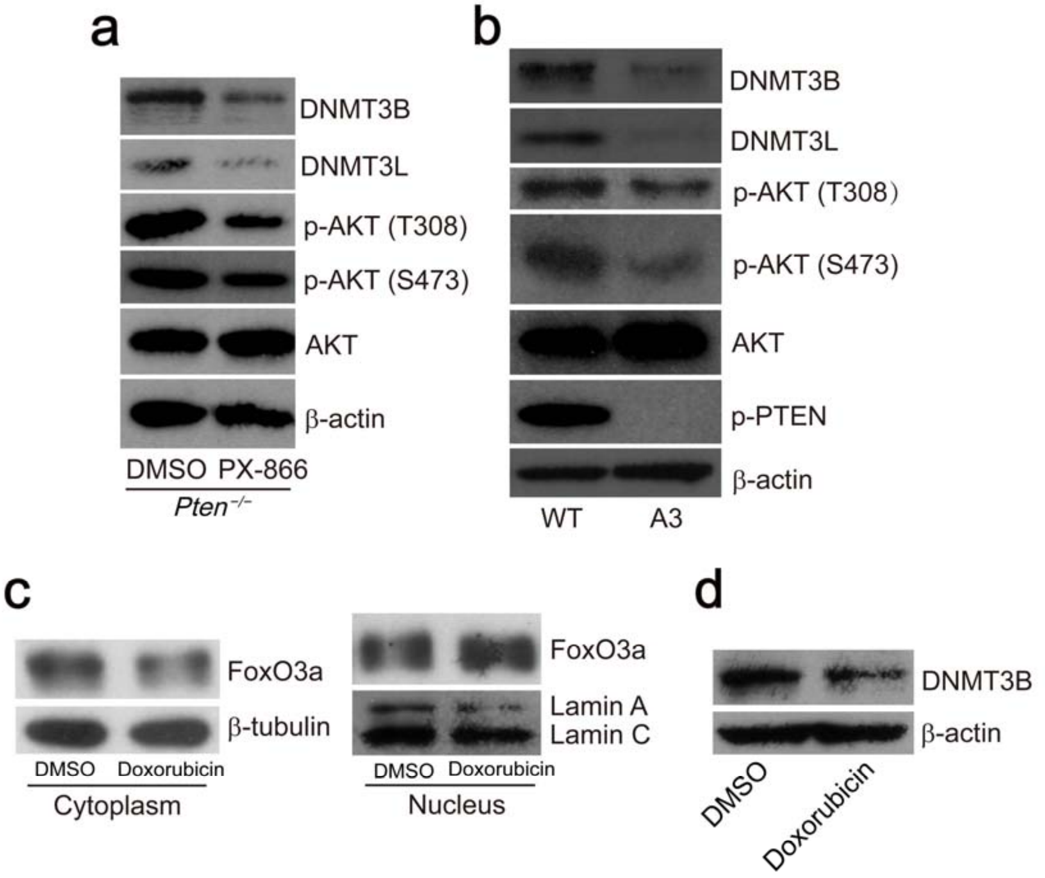
a
DNMT3B
DNMT3L
p-AKT (T308)
p-AKT (S473)
AKT
β-actin
DMSO PX-866
Pten−/−
b
DNMT3B
DNMT3L
p-AKT (T308)
p-AKT (S473)
AKT
p-PTEN
β-actin
WT A3
c
FoxO3a
β-tubulin
DMSO Doxorubicin
Cytoplasm
FoxO3a
Lamin A
Lamin C
DMSO Doxorubicin
Nucleus
d
DNMT3B
β-actin
DMSO
Doxorubicin

Figure S5

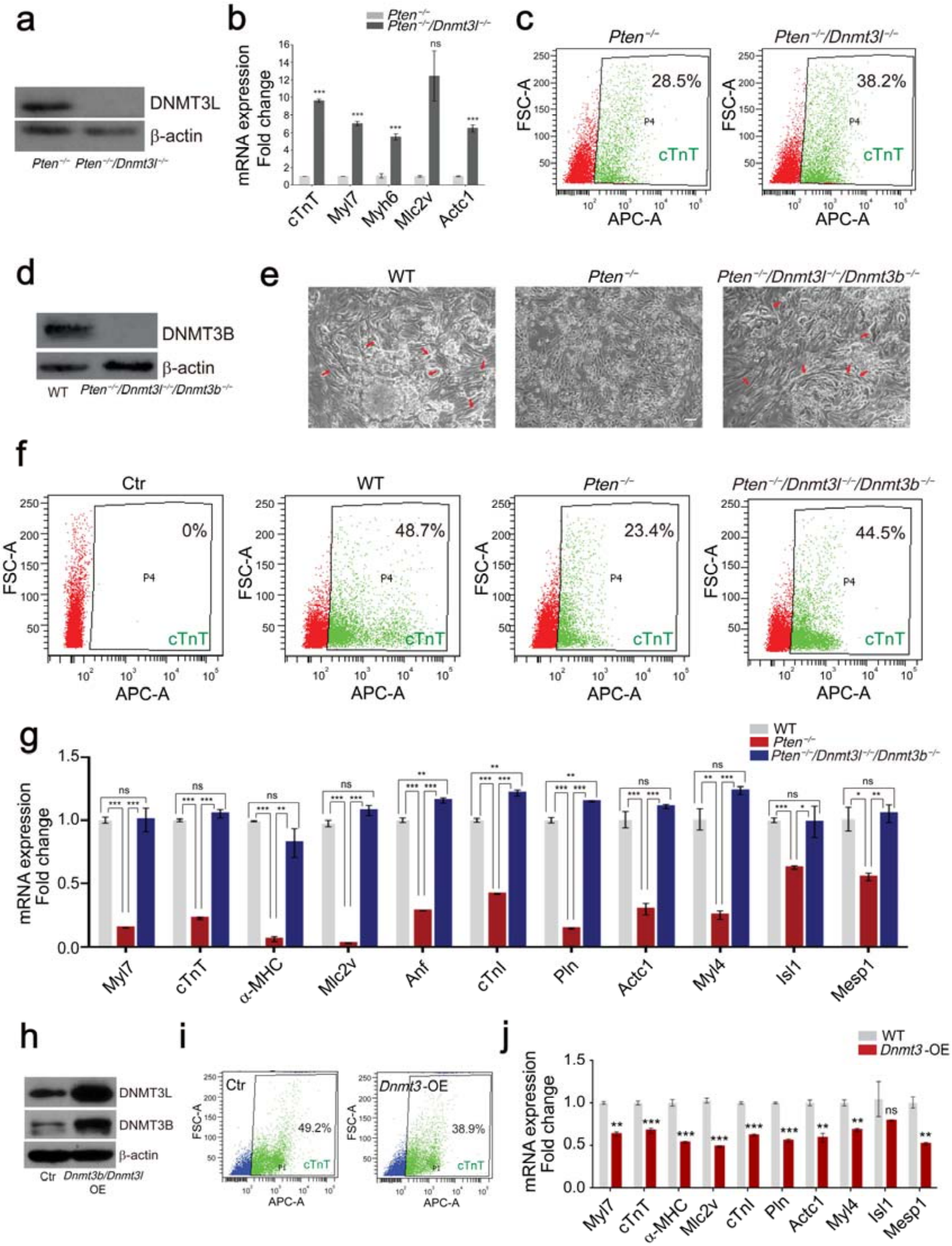

a
DNMT3L
β-actin
Pten−/− Pten−/−/Dnmt3l−/−
b
Pten−/−
Pten−/−/Dnmt3l−/−
mRNA expression Fold change
cTnT
Myl7
Myh6
Mlc2v
Actc1
c
Pten−/−
Pten−/−/Dnmt3l−/−
FSC-A
APC-A
28.5%
38.2%
cTnT
d
DNMT3B
β-actin
WT Pten−/−/Dnmt3l−/−/Dnmt3b−/−
e
WT
Pten−/−
Pten−/−/Dnmt3l−/−/Dnmt3b−/−
f
Ctr
WT
Pten−/−
Pten−/−/Dnmt3l−/−/Dnmt3b−/−
0%
48.7%
23.4%
44.5%
cTnT
FSC-A
APC-A
g
WT
Pten−/−
Pten−/−/Dnmt3l−/−/Dnmt3b−/−
mRNA expression Fold change
Myl7
cTnT
α-MHC
Mlc2v
Anf
cTnI
Pln
Actc1
Myl4
Isl1
Mesp1
h
DNMT3L
DNMT3B
β-actin
Ctr Dnmt3b/Dnmt3l OE
i
Ctr
Dnmt3-OE
49.2%
38.9%
cTnT
FSC-A
APC-A
j
WT
Dnmt3-OE
mRNA expression Fold change
Myl7
cTnT
α-MHC
Mlc2v
cTnI
Pln
Actc1
Myl4
Isl1
Mesp1

Figure S6

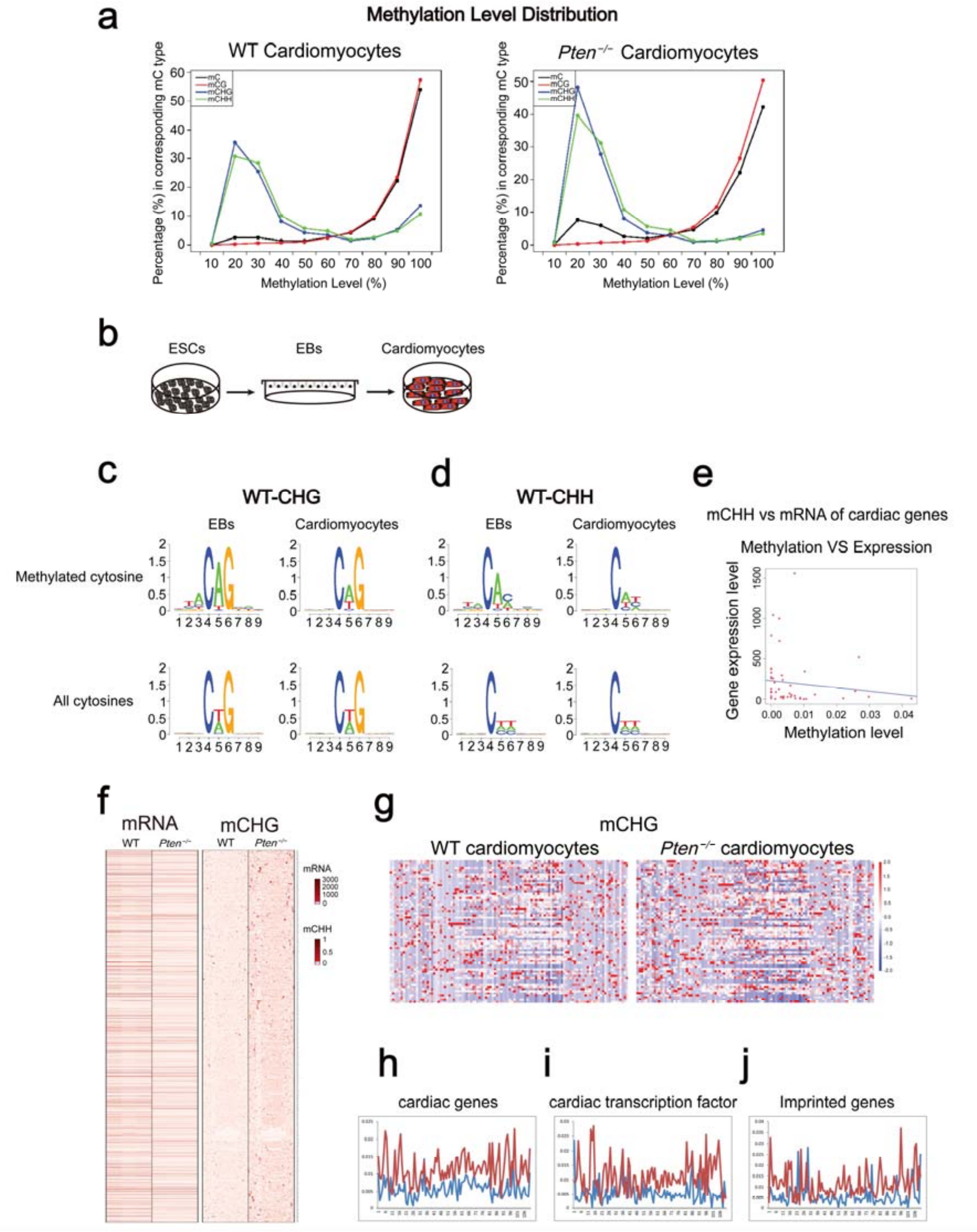

a
Methylation Level Distribution
WT Cardiomyocytes
Pten−/− Cardiomyocytes
mC
mCG
mCHG
mCHH
Percentage (%) in corresponding mC type
Methylation Level (%)
b
ESCs
EBs
Cardiomyocytes
c
WT-CHG
d
WT-CHH
EBs
Cardiomyocytes
Methylated cytosine
All cytosines
e
mCHH vs mRNA of cardiac genes
Methylation VS Expression
Gene expression level
Methylation level
f
mRNA
mCHG
WT
Pten−/−
mRNA
mCHH
g
mCHG
WT cardiomyocytes
Pten−/− cardiomyocytes
h
cardiac genes
i
cardiac transcription factor
j
Imprinted genes

Figure S7

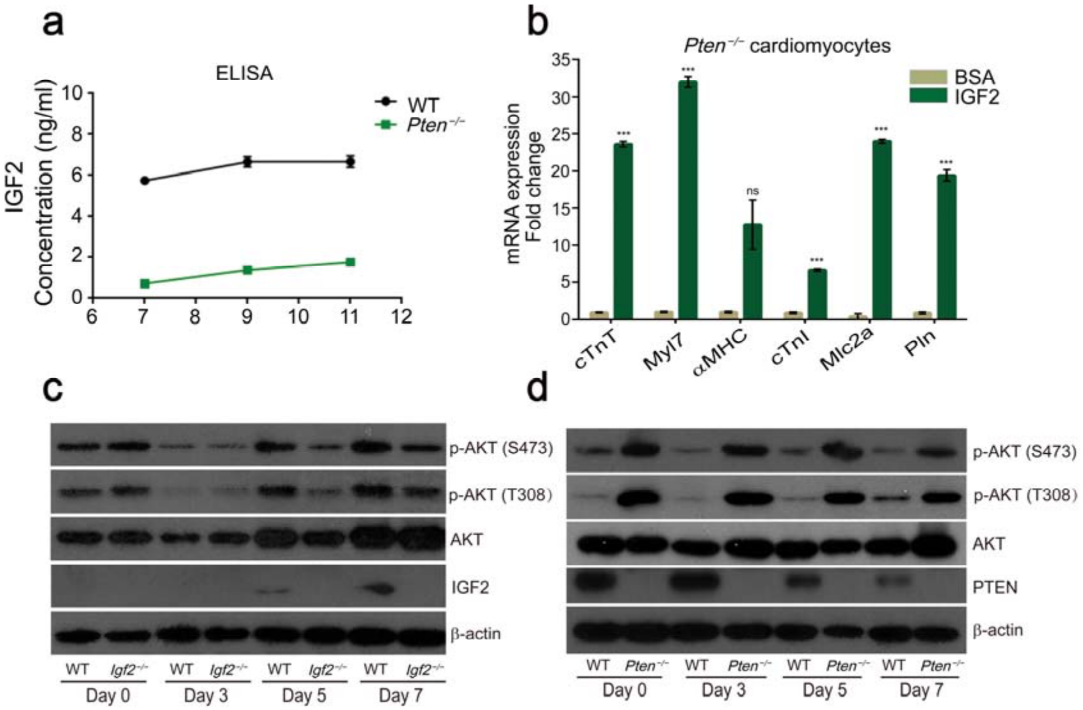

a
ELISA
IGF2
Concentration (ng/ml)
WT
Pten−/−
b
Pten−/− cardiomyocytes
mRNA expression
Fold change
BSA
IGF2
ns
cTnT
Myl7
αMHC
cTnI
Mlc2a
Pln
c
p-AKT (S473)
p-AKT (T308)
AKT
IGF2
β-actin
WT
Igf2−/−
Day 0
Day 3
Day 5
Day 7
d
p-AKT (S473)
p-AKT (T308)
AKT
PTEN
β-actin
WT
Pten−/−
Day 0
Day 3
Day 5
Day 7

Figure S8

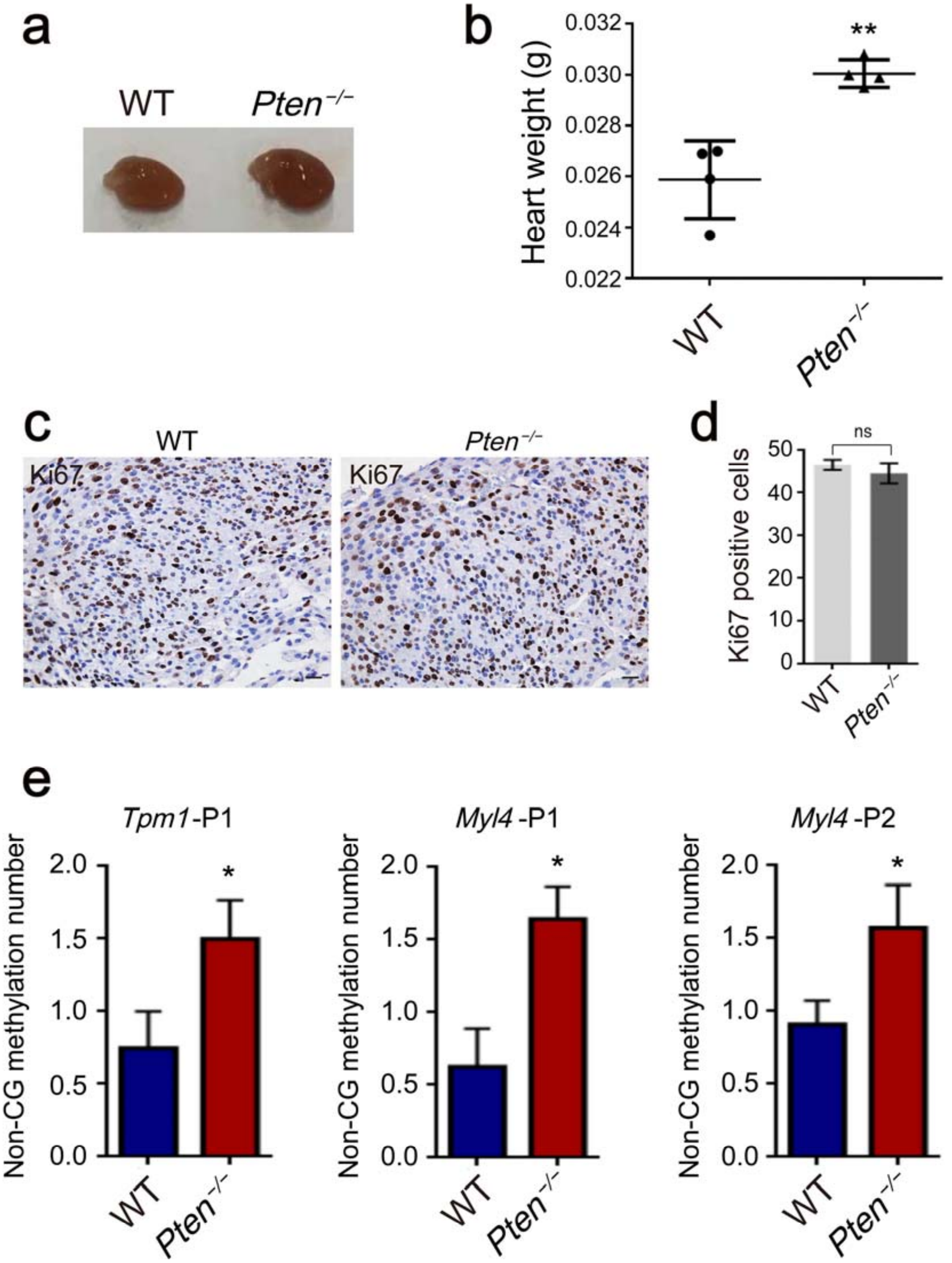
a
WT
Pten−/−
b
Heart weight (g)
0.032
0.030
0.028
0.026
0.024
0.022
**
WT
Pten−/−
c
WT
Pten−/−
Ki67
Ki67
d
Ki67 positive cells
ns
50
40
30
20
10
0
WT
Pten−/−
e
Tpm1-P1
Myl4 -P1
Myl4 -P2
Non-CG methylation number
2.0
1.5
1.0
0.5
0.0
*
WT
Pten−/−

Figure S9

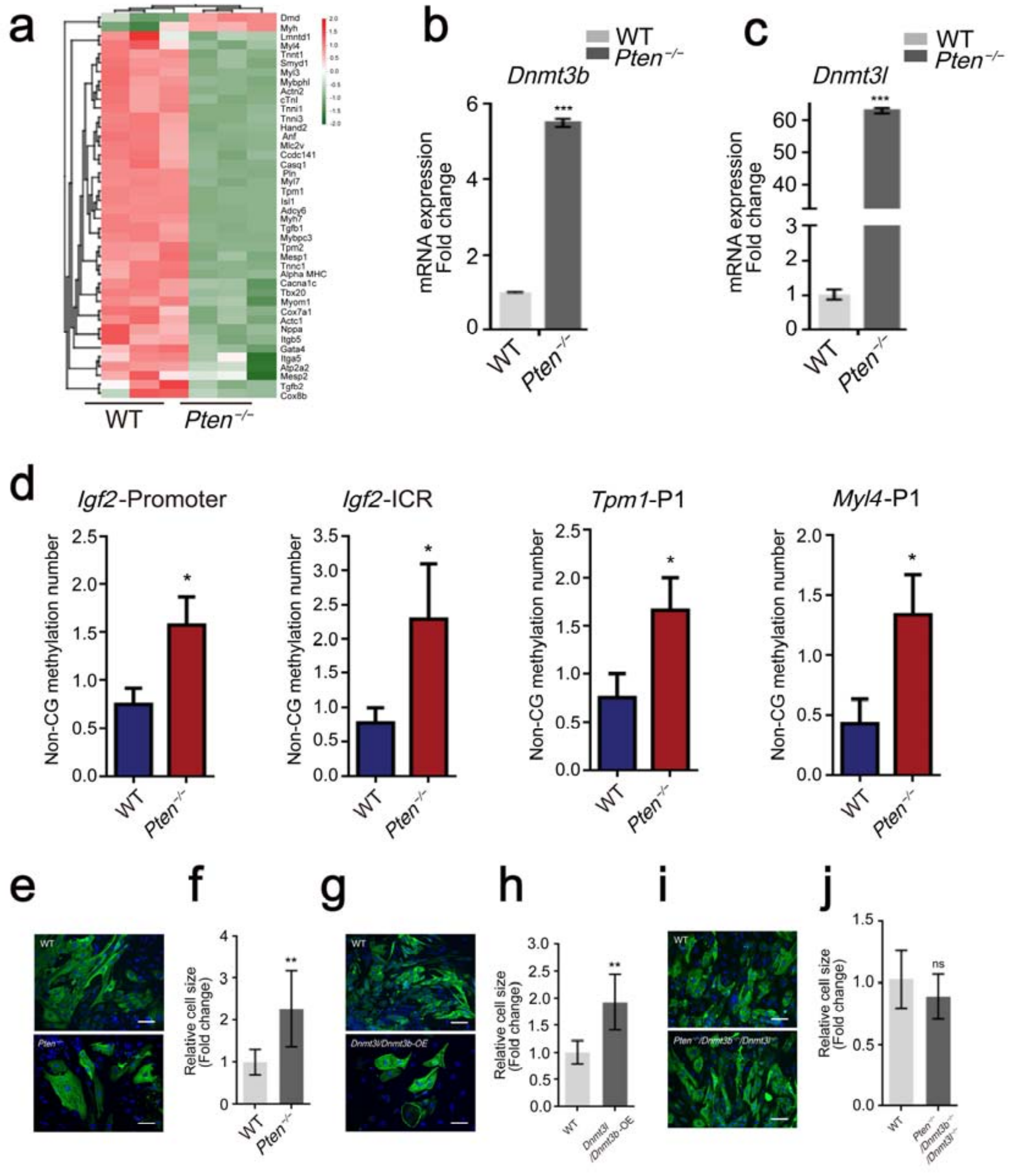
a
WT
Pten−/−
b
WT
Pten−/−
Dnmt3b
mRNA expression
Fold change
***
c
WT
Pten−/−
Dnmt3l
***
d
Igf2-Promoter
Igf2-ICR
Tpm1-P1
Myl4-P1
Non-CG methylation number
e
f
Relative cell size
(Fold change)
**
g
Dnmt3l/Dnmt3b-OE
h
**
i
j
ns

Figure S10

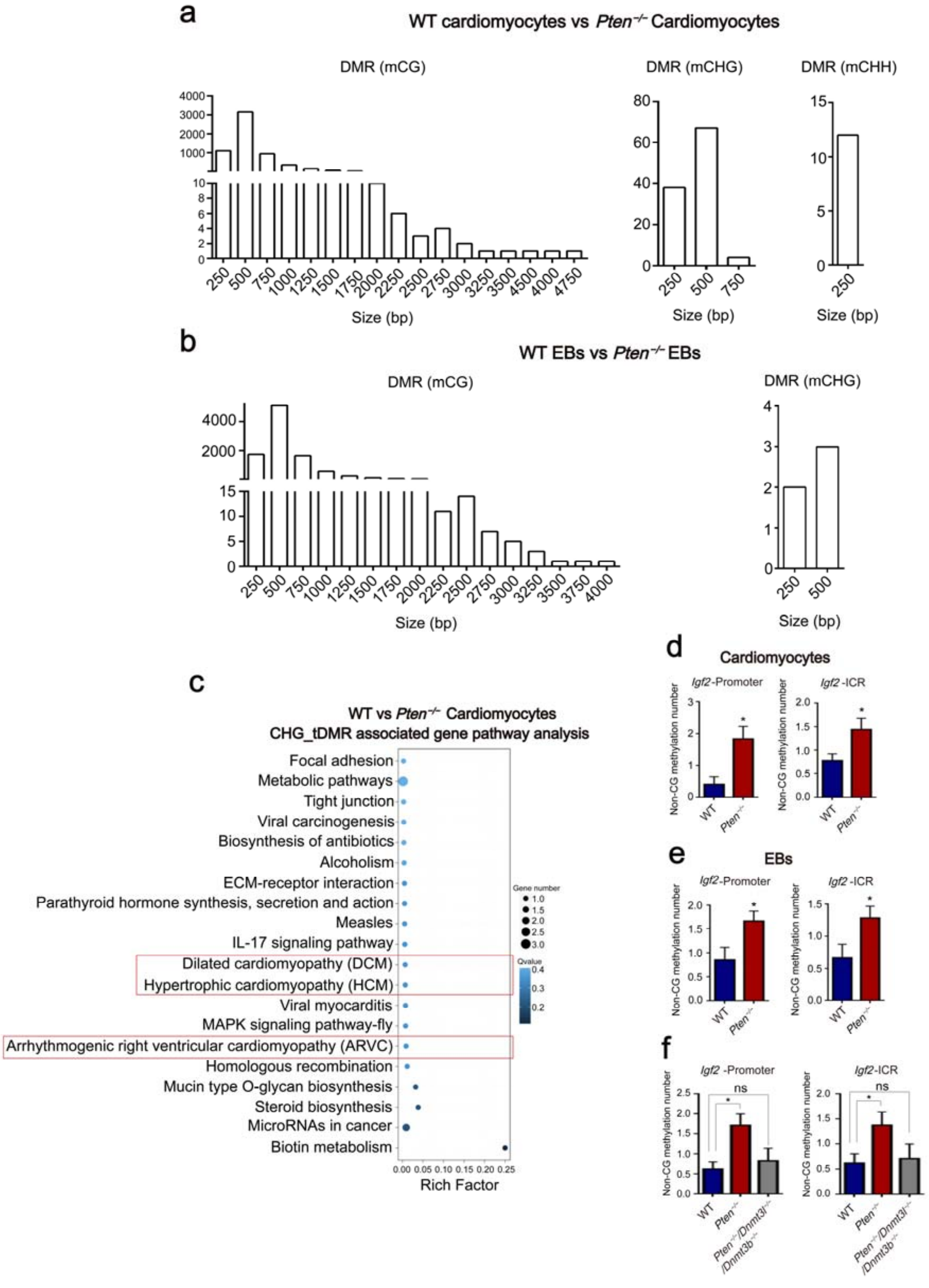
a
WT cardiomyocytes vs Pten−/− Cardiomyocytes
DMR (mCG)
DMR (mCHG)
DMR (mCHH)
Size (bp)
b
WT EBs vs Pten−/− EBs
DMR (mCG)
DMR (mCHG)
Size (bp)
c
WT vs Pten−/− Cardiomyocytes
CHG_tDMR associated gene pathway analysis
Focal adhesion
Metabolic pathways
Tight junction
Viral carcinogenesis
Biosynthesis of antibiotics
Alcoholism
ECM-receptor interaction
Parathyroid hormone synthesis, secretion and action
Measles
IL-17 signaling pathway
Dilated cardiomyopathy (DCM)
Hypertrophic cardiomyopathy (HCM)
Viral myocarditis
MAPK signaling pathway-fly
Arrhythmogenic right ventricular cardiomyopathy (ARVC)
Homologous recombination
Mucin type O-glycan biosynthesis
Steroid biosynthesis
MicroRNAs in cancer
Biotin metabolism
Gene number
Qvalue
Rich Factor
d
Cardiomyocytes
Igf2-Promoter
Igf2 -ICR
Non-CG methylation number
WT
Pten−/−
e
EBs
Igf2-Promoter
Igf2 -ICR
Non-CG methylation number
WT
Pten−/−
f
Igf2 -Promoter
Igf2-ICR
ns
Non-CG methylation number
WT
Pten−/−
Pten−/−/Dnmt3l−/−/Dnmt3b−/−

Figure S11

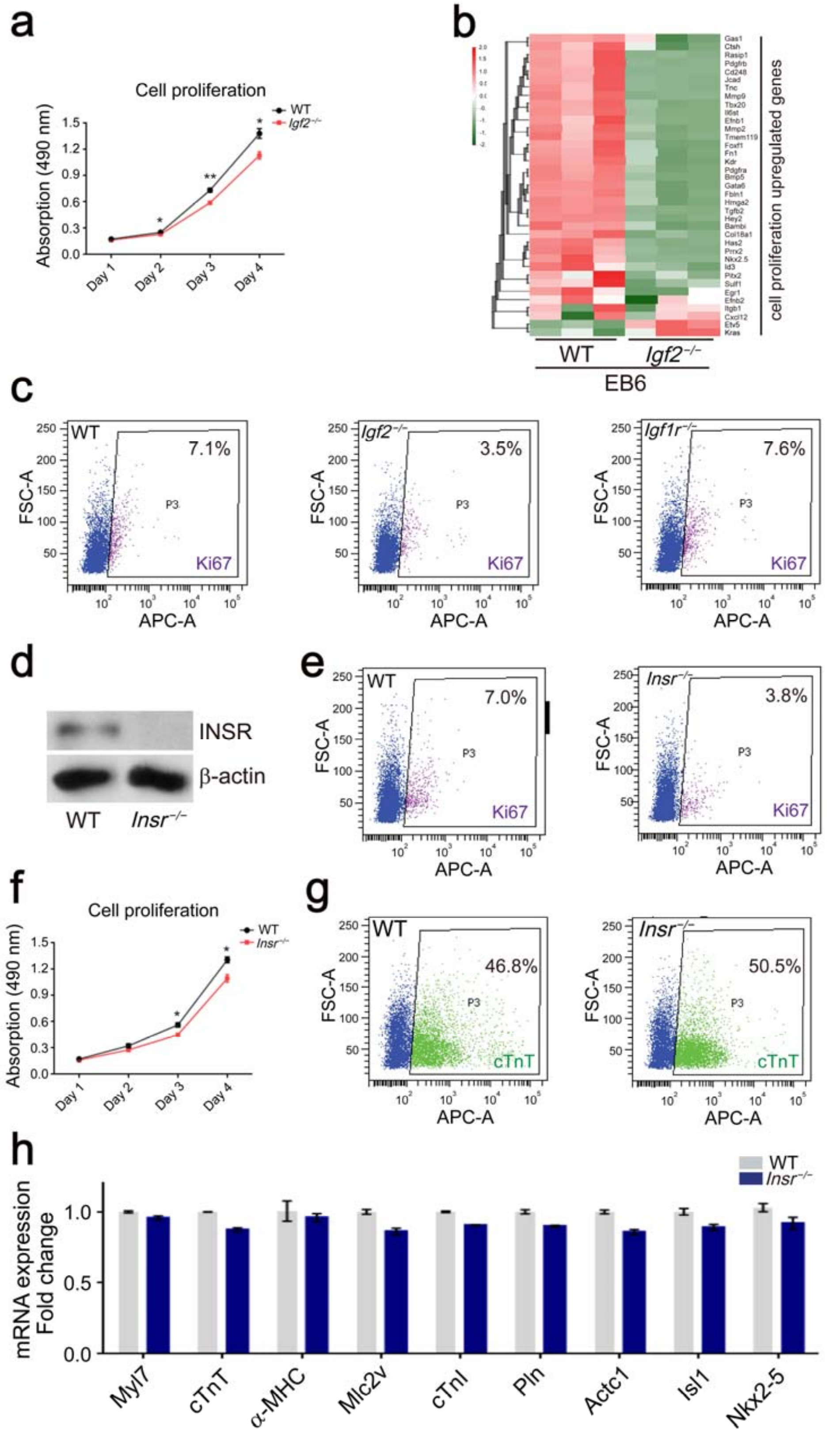
a
Cell proliferation
WT
Igf2−/−
Absorption (490 nm)
Day 1
Day 2
Day 3
Day 4
b
cell proliferation upregulated genes
WT
Igf2−/−
EB6
c
WT
7.1%
Igf2−/−
3.5%
Igf1r−/−
7.6%
FSC-A
APC-A
P3
Ki67
d
INSR
β-actin
WT
Insr−/−
e
WT
7.0%
Insr−/−
3.8%
f
Cell proliferation
WT
Insr−/−
Absorption (490 nm)
g
WT
46.8%
Insr−/−
50.5%
cTnT
h
WT
Insr−/−
mRNA expression
Fold change
Myl7
cTnT
α-MHC
Mlc2v
cTnI
Pln
Actc1
Isl1
Nkx2-5
